# Modulation of the PI3K/Akt signaling pathway by steroidal saponins: therapeutic implications in cancer

**DOI:** 10.3389/fphar.2026.1779073

**Published:** 2026-04-29

**Authors:** Mohammad Bagher Majnooni, Maryam Naseri, Shayan Bakhshy-Chenary, Younes Zeinalii, Mahdis Azadi, Mohammad Hosein Farzaei, Sajad Fakhri, Syed Mustafa Ghanadian, Javier Echeverría

**Affiliations:** 1 Pharmaceutical Sciences Research Center, Health Institute, Kermanshah University of Medical Sciences, Kermanshah, Iran; 2 Student Research Committee, School of Pharmacy, Kermanshah University of Medical Sciences, Kermanshah, Iran; 3 Student Research Committee, School of Medicine, Kermanshah University of Medical Sciences, Kermanshah, Iran; 4 Department of Pharmacognosy, Isfahan Pharmaceutical Sciences Research Center, School of Pharmacy and Pharmaceutical Sciences, Isfahan University of Medical Sciences, Isfahan, Iran; 5 Departamento de Ciencias del Ambiente, Facultad de Química y Biología, Universidad de Santiago de Chile, Santiago, Chile

**Keywords:** angiogenesis inhibition, anticancer activity, apoptosis, multidrug resistance, PI3K/AKT signaling, steroidal saponins

## Abstract

**Background:**

The phosphatidylinositol 3-kinase/protein kinase B (PI3K/Akt) pathway is considered essential for cancer progression and the regulation of cellular processes, including proliferation, survival, metastasis, and angiogenesis. Conventional therapies using targeted agents such as alpelisib and everolimus have limited the effectiveness of inhibitors in exploiting effective resistance mechanisms. Steroidal saponins (SSs) are a diverse group of natural compounds recognized as anticancer agents that target multiple cells and pathways.

**Purpose:**

To synthesize current evidence on how SSs modulate the PI3K/Akt pathway to produce anticancer effects and to outline translational opportunities and limitations.

**Methods:**

We conducted a review of preclinical and translational studies indexed in PubMed/Scopus/Google Scholar up to August 2025. Studies were included if they reported mechanistic or functional modulation of the PI3K/Akt/mTOR axis (*in vitro*, *in vivo*, or *ex vivo*). Relevant data were compiled and organized based on compound class, experimental model, dose/exposure, and PI3K/Akt-related molecular readouts.

**Results and Discussion:**

This review highlights the potential of SSs to target the PI3K/Akt pathway and combat cancer progression, and addresses the limitations of conventional therapies in overcoming therapeutic resistance. SSs, as small-molecule phytochemicals, exert anticancer effects through the induction of apoptosis, inhibition of metastasis and angiogenesis, alteration of the tumor microenvironment to therapeutic advantage, promotion of the immune response, and other mechanisms that reverse multidrug resistance by modulating the PI3K/Akt pathway.

**Conclusion:**

The combination of SSs with chemotherapeutic agents, given emerging preclinical evidence of small-molecule efficacy, supports the development of new anticancer therapies. However, the development of SSs for clinical use remains limited due to their low bioavailability, systemic toxicity, and lack of target specificity.

## 1 Introduction

Currently, the development of therapeutic methods for managing cancer, which kills thousands of people around the world, is still one of the most significant challenges in healthcare (Schwartz, 2024; Siegel et al., 2024). Meanwhile, cancer researchers have placed substantial emphasis on natural products and their secondary metabolites from plants, marine organisms, and microorganisms, which have long been a viable and sensible source for the discovery of anticancer drug molecules. Because of these efforts, anti-cancer drugs have been developed, such as paclitaxel and its analogs (a terpenoid alkaloid isolated from *Taxus brevifolia* Nutt. [Taxaceae]) (Sousa-Pimenta et al., 2023); vinblastine, vincristine, and their related analogs (alkaloids isolated from *Catharanthus roseus* (L.) G.Don [Apocynaceae]) (Mendonce et al., 2025); camptothecin and its associated analogs (an alkaloid from *Camptotheca acuminata* Decne. [Nyssaceae]) (Sriram et al., 2005); podophyllotoxin and its related analogs (lignin derivatives isolated from *Podophyllum* spp. [Berberidaceae]) (Jin et al., 2022); and anthracyclines like doxorubicin (purified from a *Streptomyces peucetius* [Streptomycetaceae]) (Mattioli et al., 2023), that are routinely used for chemotherapy in various cancers.

The unique chemical structure of steroidal saponins (SSs), primarily composed of a 27-carbon skeleton with/without a heterocyclic ring, often attached to a sugar chain, underpins their diverse pharmacological and biological effects, including cytotoxic and anticancer properties. SSs inhibit various cancers by targeting multiple cellular signaling mechanisms involved in cancer cell growth, proliferation, angiogenesis, metastasis, and migration, such as Janus kinase/Signal transducer and activator of transcription (JAK/STAT), human epidermal growth factor receptor 2 (HER2), vascular endothelial growth factor/vascular endothelial growth factor receptor (VEGF/VEGFR), matrix metalloproteinase 9/matrix metalloproteinase 2 (MMP9/MMP2), and tumor protein p53 (p53), as well as by suppressing inflammatory pathways like mitogen-activated protein kinases (MAPKs) and nuclear factor kappa-light-chain-enhancer of activated B cells (NF-κB) (Elekofehinti et al., 2021; Bouabdallah et al., 2023; Majnooni et al., 2023).

The phosphoinositide 3-kinase/Protein kinase B (PI3K/Akt) pathway is a fundamental cellular pathway that regulates numerous primary cellular functions in cancer, including cell growth, survival, invasion, and angiogenesis. This pathway is dysregulated in many cancers, including breast, colorectal, gastric, and prostate. In cancers, dysregulation of this pathway enhances angiogenesis by activating VEGF and promotes epithelial-mesenchymal transition (EMT), leading to metastatic spread. Also, in cancer stem cells (CSCs), it is a key contributor to maintaining tumor heterogeneity and invasiveness. Because of its central role in cancer, the PI3K/Akt signaling pathway is a promising target for drug development. Several specific inhibitors, such as alpelisib and everolimus, that target PI3K isoforms have demonstrated clinical utility in cancer treatment (Fresno Vara et al., 2004; Rascio et al., 2021; Xue et al., 2021; Karami Fath et al., 2022; Yu et al., 2022). There are also other PI3K/Akt inhibitors, such as samotolisib (LY3023414), that are in clinical trials for the treatment of various cancers (Hong et al., 2021; Sweeney et al., 2022).

Several review articles have summarized the anticancer effects of saponins, particularly SSs, and their ability to regulate various cellular signaling pathways. While recent reviews have discussed the involvement of SSs in major oncogenic signaling pathways, their treatment of PI3K/Akt signaling has generally been presented as part of a broader multi-pathway overview rather than as a dedicated, mechanistically integrated analysis. In particular, limited emphasis has been placed on systematically organizing available evidence according to (i) structural classes of SSs, (ii) specific nodes within the PI3K/Akt cascade (e.g., upstream receptor modulation, PI3K activation, Akt phosphorylation, or downstream mTOR signaling), and (iii) the experimental context (*in vitro* vs. *in vivo* models and dosage considerations). Furthermore, the question of potential isoform-selective interactions within the PI3K family remains insufficiently clarified in the current literature.

In contrast, the present review places the PI3K/Akt pathway at the center of SS therapy in cancer. It systematically compiles evidence on how SSs from various botanical sources and structural classes utilize the PI3K/Akt pathway to exert their antitumor effects. Rather than surveying all signaling pathways equally, we specifically synthesize and compare mechanistic data on PI3K/Akt modulation, highlight recurring molecular patterns across SS subclasses, and identify unresolved questions—including structural determinants and possible isoform-specific effects—that warrant further investigation.

In addition, this review describes the molecular mechanisms of action, chemical structure, natural sources, pharmacological and biological effects of the SSs, effective dosages, *in vitro*/*in vivo* models, and combination therapy options, thus providing a focused and integrated overview of the therapeutic potential of SSs targeting the PI3K/Akt signaling pathway in cancer (Elekofehinti et al., 2021; Porte et al., 2022; Bouabdallah et al., 2023; Zhu et al., 2023; Cui et al., 2024; Wang F. et al., 2024). These findings can inform more extensive preclinical and clinical studies of SSs as potential anticancer agents.

## 2 PI3K/Akt signaling pathway in cancer etiology

The PI3K/Akt/mTOR pathway is essential for regulating multiple cellular processes. Genetic mutations and alterations, as exemplified by p110α subunit of PI3K (PIK3CA) mutations or phosphatase and tensin homolog (PTEN) loss, cause constitutive activation of the PI3K/Akt/mTOR pathway, which supports cancer cell growth and survival, thus contributing to tumor formation (Fresno Vara et al., 2004; Jiang et al., 2020; Rascio et al., 2021; Xue et al., 2021; Yu et al., 2022). Further interaction with other pathways, such as the RAS/RAF/MEK/ERK pathway, can enhance its oncogenic activity and influence tumor growth (Karami Fath et al., 2022; Khezri et al., 2022).

**FIGURE 1:**
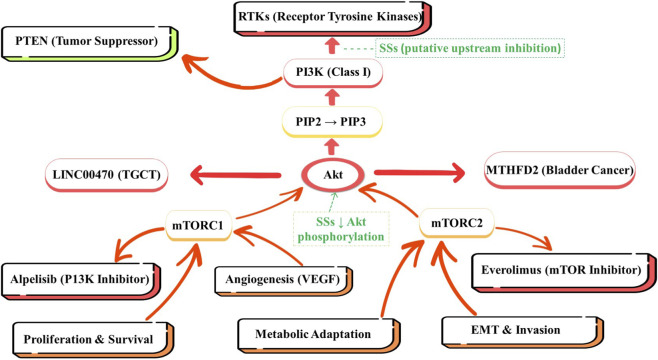
Enhanced PI3K/Akt signaling pathway in cancer etiology and hypothesized sites of intervention by steroidal saponins (SSs). The figure depicts key molecular components and processes regulated by Akt, including phosphorylation of BAD and FOXO transcription factors, which promote cell survival and proliferation through mTORC1-directed protein synthesis. Dashed arrows indicate putative inhibitory actions of SSs at specific nodes of the pathway, including upstream modulation of receptor tyrosine kinases (RTKs) and suppression of Akt phosphorylation. Akt: Protein Kinase B, Alpelisib: PI3K Inhibitor, EMT: Epithelial-Mesenchymal Transition, Everolimus: mTOR Inhibitor, LINC00470: Long Intergenic Non-Coding RNA 00470, MTHFD2: Methylenetetrahydrofolate Dehydrogenase 2, mTORC1: Mechanistic Target of Rapamycin Complex 1, mTORC2: Mechanistic Target of Rapamycin Complex 2, PI3K: Phosphatidylinositol 3-Kinase (Class I), PIP2: Phosphatidylinositol 4,5-bisphosphate, PIP3: Phosphatidylinositol 3,4,5-trisphosphate, PTEN: Phosphatase and Tensin Homolog (Tumor Suppressor), RTKs: Receptor Tyrosine Kinases, TGCT: Testicular Germ Cell Tumor, VEGF: Vascular Endothelial Growth Factor.

The PI3K/Akt/mTOR pathway integrates upstream inputs, including receptor tyrosine kinases (RTKs), G-protein-coupled receptors (GPCRs), and PTEN, as well as other factors, to ultimately regulate several key downstream processes, as illustrated in Figure 1. This includes processes such as cellular proliferation, survival, epithelial-mesenchymal transition (EMT), and angiogenesis, as well as other crucial downstream signaling (Zhu et al., 2011; Fruman et al., 2017; Zhang et al., 2017). Figure 1 provides a basic visual representation of the PI3K/Akt/mTOR pathway’s role in cancer etiology and its potential for therapeutic targeting.

### 2.1 Role in cancer progression

The PI3K/Akt/mTOR signaling pathway plays a crucial role in regulating cell cycle progression and inhibiting apoptosis in cancer cells. Due to the activation of this pathway, cell-cycle progression occurs through the mechanistic target of rapamycin complex 1 (mTORC1)-mediated activation of protein synthesis, leading to cell growth (Fresno Vara et al., 2004; Yang et al., 2019; Rascio et al., 2021; Yu et al., 2022). Akt activation leads to the phosphorylation of several regulators, including Bcl-2-associated death promoter (BAD) and Forkhead box O (FOXO) transcription factors, which are negative regulators of apoptosis and cell survival (Hai-zhong et al., 2022). Figure 1 illustrates how Akt phosphorylation of the BAD and FOXO transcription factors promotes cell survival and proliferation by activating mTORC1-directed protein synthesis and cell growth.

In gastric cancer, the oncogene Fam198b activates the PI3K/Akt/mTOR and anti-apoptotic signaling pathways, enabling cancer cells to proliferate (Chen et al., 2024). CircFGFR1int2 in prostate cancer also supports this idea, as it enhances fibroblast growth factor receptor 1 (FGFR1) signaling, thereby inducing tumor growth. This suggests that some oncogenes utilize the PI3K/Akt/mTOR pathway to promote tumor aggressiveness (Wang R. et al., 2023).

The PI3K/Akt/mTOR pathway is involved in angiogenesis, in which VEGF signaling promotes angiogenesis and supports blood flow to rapidly replicating tumors. In the case of colorectal cancer, VEGF expression is increased in a hyperglycemic state and therefore increases vascularization and tumor growth (Ding et al., 2024). Lastly, the PI3K/Akt/mTOR signaling pathway mediates the epithelial-to-mesenchymal transition process, converting epithelial cells into mesenchymal-like cells with increased migratory and invasive potential.

mTORC1, as shown in Figure 1, positively regulates the upregulation of VEGF to promote angiogenesis, while mTORC2 is responsible for EMT, which involves the transformation of epithelial cells into invasive mesenchymal phenotypes. In testicular germ cell tumors (TGCTs), the EMT process that long intergenic non-protein coding RNA 470 (LINC00470) engages with promotes epithelial-to-mesenchymal transition and facilitates cell movement and dispersion (Liu et al., 2024). Similarly, peroxisomal membrane protein 4 (PXMP4), although not directly linked to mTORC1 or mTORC2, modulates upstream PI3K/Akt signaling activators that promote EMT in gastric cancer, underscoring its important role in cell motility and invasion. The PI3K/Akt/mTOR signaling pathway is renowned for its role in regulating cell metabolism, one of the core hallmarks of cancer, by promoting the biosynthesis of glucose and lipids to meet the energy requirements of rapidly dividing cells (Rascio et al., 2021; Karami Fath et al., 2022; Glaviano et al., 2023). In colorectal cancer, hyperglycemia has been shown to inhibit autophagy and promote expression of EMT markers, such as N-cadherin and Zinc finger E-box-binding homeobox 1 (ZEB1), thereby promoting cancer growth (Ding et al., 2024). These observations highlight the metabolic profile of cancer cells and, consequently, their dependence on the PI3K/Akt/mTOR signaling pathway.

Figure 1 illustrates how mTOR signaling mediates metabolic adaptation in cancer through increased glucose and lipid biosynthesis during the rapid proliferation of tumor cells. Methylenetetrahydrofolate dehydrogenase 2 (MTHFD2) acts as a tumor suppressor in bladder cancer; however, when overexpressed, it can activate this pathway by upregulating programmed death-ligand 1 (PD-L1) and contributing to chemotherapy resistance (Deng et al., 2022). Conversely, metabolic stress in the tumor microenvironment utilizes mTOR signaling to incorporate nutrient stress, thus enabling cancer cells to adapt and survive, reaffirming the notion of metabolic reprogramming in cancer.

Several tumor-specific factors promote cancer cell proliferation by regulating the PI3K/Akt/mTOR pathway. For example, curcumin indirectly targets the PI3K/Akt/mTOR pathway in gastric cancer patients by downregulating the long non-coding RNA AC022424.2, thereby inhibiting proliferation and invasion through induction of apoptosis (Wang B.-S. et al., 2024). In addition, miRNA-766-3p targets the PI3K/Akt/mTOR pathway by downregulating collagen 1A1 (COL1A1), demonstrating the potential use of miRNAs in cancer chemotherapies (Ding et al., 2024).

In epithelial ovarian cancer (EOC), tumor necrosis factor α-induced protein-8-like-2 (TIPE2) functions as a tumor suppressor by downregulating the PI3K/Akt/mTOR signaling pathway, thereby regulating the immune response and preventing tumor progression (Xu et al., 2023). Lastly, evidence in mouse spermatogonia suggests that microcystin-LR (MC-LR) activates the PI3K/Akt/mTOR pathway, driving malignant cell proliferation and linking environmental contaminants to cancer (Du et al., 2023).

### 2.2 Therapeutic implications

The PI3K/Akt/mTOR signaling pathway is a primary target for cancer treatment, and inhibitors have been developed to block pathway activation. Isoform-specific inhibitors, such as alpelisib, block the PI3K catalytic subunits, while everolimus directly blocks the mTOR complex (Fresno Vara et al., 2004; Mayer and Arteaga, 2016; Yang et al., 2019; Rascio et al., 2021; Xue et al., 2021; Karami Fath et al., 2022; Yu et al., 2022). The effectiveness of these therapies has generated clinical treatment progress for breast cancer and gastric cancer by blocking growth and inducing apoptosis. As depicted in Figure 1, inhibitors can act at different nodes in the pathway to disrupt tumor growth and progression. However, there are pathways of resistance or multiple feedback mechanisms that limit the efficacy of these therapies. One of these mechanisms involves reactivating the PI3K/Akt/mTOR signaling pathway or other signaling pathways, such as the MAPK pathway. This reactivation limits the long-term effectiveness of these therapies, suggesting the development of combination therapies that block multiple pathways (Fresno Vara et al., 2004; Yang et al., 2019; Yu et al., 2022). A few novel strategies focus on blocking with less drug resistance. For example, the PI3K inhibitor LY294002 has been shown to inhibit EMT, as determined by PXMP4, in gastric cancer, demonstrating potential for more specific therapies (Li et al., 2024). Furthermore, an emerging therapeutic approach targeting SPRED3 in thyroid carcinoma has been identified, acting as an intrinsic regulator that suppresses the PI3K/Akt signaling pathway and establishing it as a novel target (Zhang X. et al., 2024).

Resistance to PI3K/Akt/mTOR-targeted therapies represents the next significant challenge for TGCT. These resistance mechanisms are multifactorial and involve the overexpression of multiple genes, including LINC00470, which reduces TGCTs’ sensitivity to chemotherapy (Liu et al., 2024). In a similar example, bladder cancer overexpresses MTHFD2, which promotes PD-L1 expression, a gene that mediates immune tolerance to chemotherapy (Deng et al., 2022). In Figure 1, we present examples of resistance mechanisms that culminate in pathway reactivation or compensatory MAPK signaling, which underlie the rationale for using combination therapy to block two or more distinct therapeutic nodes. Combination therapy is effective at overcoming resistance mechanisms. Curcumin, a natural product that diminishes PI3K/Akt signalling, has demonstrated superior treatment outcomes in gastric cancer when combined with conventional therapeutic regimens (Wang B.-S. et al., 2024). This approach targets primary and back-up signaling pathways to suppress tumors more effectively.

New therapeutic options, such as Ag@ZnO nanocomposites used as nanoparticles, have demonstrated both direct/indirect activity via the PI3K/Akt/mTOR signaling pathway, mediated by oxidative stress and apoptosis (Yin et al., 2024). These domains represent a novel, environmentally sustainable approach to chemotherapy agents for cervical and colorectal cancers. Furthermore, the upregulation of VEGF and mediators of apoptosis has been observed with the use of *Astragalus mongholicus* Bunge (syn. *Astragalus membranaceus*) [Fabaceae] and Radix paeoniae rubra extracts, providing evidence-based links between metabolism and pathway inhibition (Jiang et al., 2023). These new therapies could provide a useful pathway for cancer inhibition, complemented by further systemic metabolic approaches and immune-oncology strategies.

The PI3K/Akt/mTOR pathway influences tumor growth and metastasis but also impairs normal metabolic processes, prevents normal cell death, and contributes to systemic diseases associated with cancer. Figure 1 demonstrates that this pathway involves not only signaling for tumor growth and metastasis but also immune escape (indirectly mediated by PD-L1 upregulation), metabolic alterations, and the requirement for sustained angiogenesis, thereby reaffirming that tumor progression is a systemic process. This pathway regulates metabolism, altering glucose uptake, lipid synthesis, and ATP production to sustain tumor growth. In papillary thyroid carcinoma, metabolic factors and the PI3K/Akt pathway interact to promote tumor cell proliferation (Yang et al., 2023). In hepatocellular carcinoma (HCC), specific miRNAs, such as miR-214-3p, regulate metabolism-related pathways linked to the PI3K/Akt pathway, thereby promoting tumor progression (El-shqnqery. This pathway is also well known for fostering immune avoidance. For example, MTHFD2 in bladder cancer promotes PD-L1 and leads to non-response to immunotherapy (Deng et al., 2022). Additionally, evidence suggests that the PI3K/Akt pathway can promote angiogenesis by stimulating pro-angiogenic factors, such as VEGF, thereby sustaining tumor vascularization and growth (Jiang et al., 2023).

In more severe cases of tumor growth, the cancer cells encounter resistance to therapy. For example, TERT promoter mutations enhance PI3K/Akt activation, leading to treatment resistance in thyroid cancers (Landa et al., 2023). This presents a challenge, as we must consider various mechanisms of action in strategic target selection, including targeting the main pathway, targeting downstream effects, or a combination of both. The PI3K/Akt/mTOR signaling pathway is a major regulator of cell growth, proliferation, survival, angiogenesis, and evasion of apoptosis, as well as immune evasion. The dysregulated state of this pathway, resulting from genetic changes and alterations to the tumor microenvironment, presents an opportunity for targeted therapy.

Evidence that this pathway affects cancer metabolism and immune evasion demonstrates that dysfunctional strategies exert multiple effects on cancer. Further clarification of our current understanding of the mechanisms underlying its dysfunctional outcomes and the development of new management strategies will be critical to the future development of precision oncology.

## 3 Steroidal saponins: chemical structures and natural sources

**FIGURE 2:**
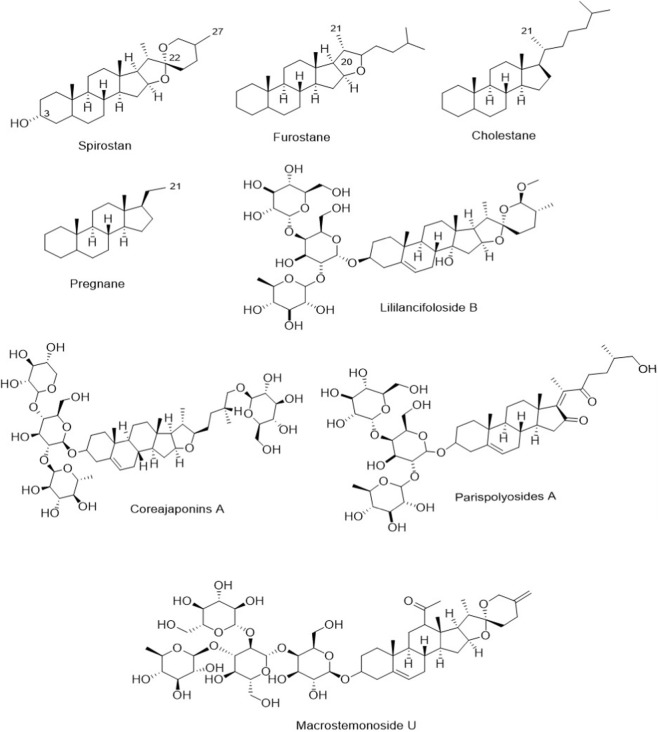
Chemical structure of some SSs. A-24: Steroidal saponin isolated from *Allium chinense*, Deltonin: Steroidal saponin derived from *Dioscorea* spp., Dioscin: Steroidal saponin isolated from *Dioscorea* spp. and *Allium* spp., Methyl protodioscin: Furostane-type steroidal saponin from *Dioscorea collettii*, OSW-1: Cholestane-type steroidal saponin from *Ornithogalum saundersiae*, PSS: Paris Steroidal Saponin, isolated from *Paris polyphylla*, , RCE-4: *Reineckia carnea* extract compound 4, Spicatoside A: Steroidal saponin isolated from *Liriope platyphylla*, TAIIISS: Timosaponin AIII steroidal saponin from *Anemarrhena asphodeloides* and *Asparagus* spp.

Saponins have been identified and isolated from natural sources, particularly plants and marine organisms, in three distinct chemical structures: triterpenoid saponins, steroidal saponins, and alkaloid saponins (Chen et al., 2021). Steroidal saponins (SSs), like other saponin chemical structures, comprise two parts, polar and nonpolar. Their nonpolar parts are 27 carbons, which are classified based on the presence of heterocyclic rings, including pyranose (spirostans, with six rings), furanose (furostanes, with five rings), and without heterocyclic rings (cholestanes and pregnanes with four rings). Spirostans are the most abundant SS in herbs. In most SS structures isolated from natural sources, carbon 3 (C-3, Figure 2) is attached to a hydroxyl group (Rothman et al., 1952; Sobolewska et al., 2016; Porte et al., 2022; Li et al., 2023). Additionally, spirostans SS are classified as monodesmodic, bisdesmodic, tridesmodic, or tetradesmodic based on the number of sugar chains attached to their aglycone. These sugar chains (mainly glucose and rhamnose) are usually attached to hydroxyl groups at C1, C3, C24, and C26 (El Aziz et al., 2019). Importantly, variations in the aglycone backbone and glycosylation patterns are not merely structural features; accumulating evidence suggests that these differences may influence membrane interactions, receptor binding, and intracellular signaling modulation—including effects on upstream regulators of the PI3K/Akt axis (Böttger and Melzig, 2013; de Groot and Müller-Goymann, 2016; Efimova and Ostroumova, 2021). Therefore, understanding the structural diversity of SSs provides a necessary framework for interpreting their differential capacity to interfere with PI3K/Akt-driven oncogenic signaling.

SS are mainly found in monocotyledonous plants and are biosynthesized via the mevalonate pathway. These saponins are biosynthesized primarily in plants and marine organisms to defend against pathogens and herbivores. Amaryllidaceae, Asparagaceae, Cucumariidae, Dioscoreaceae, Liliaceae, Melanthiaceae, and Quillajaceae are the prominent families from which SSs have been isolated (Fagbohun et al., 2023; Li et al., 2023). However, SS has been isolated from some dicotyledonous plants such as *Trigonella foenum-graecum* L. [Fabaceae] (Petit et al., 1995). New SS, including three cholestane and one pregnane, were isolated from the roots of *Dracaena cambodiana* Pierre ex Gagnep. [Asparagaceae] (Yen et al., 2024). Also, five new SSs, macrostemonoside U-Y (Figure 2), were isolated from *Allium macrostemon* Bunge [Amaryllidaceae] (Wu et al., 2024a). Four new SS, Lililancifoloside (B-E, Figure 2), isolated from the bulbs of *Lilium lancifolium* Thunb. [Liliaceae] (Zhou et al., 2024b). Recent phytochemical investigations continue to expand the catalog of naturally occurring SSs across these families, particularly in *Dioscorea*, *Allium*, *Lilium*, *Asparagus*, and *Paris* species. Several of these newly characterized spirostan and furostan derivatives have subsequently been evaluated for anticancer activity, with selected compounds demonstrating modulation of PI3K/Akt/mTOR signaling in cellular and xenograft models (Bouabdallah et al., 2023; Wang F. et al., 2024). Asparacochioside A is a novel furostanol saponin, which was identified in the root of *Asparagus cochinchinensis* (Lour.) Merr. [Asparagaceae] (Kim et al., 2021). Coreajaponins A (Figure 2) and B, as two furostanols, were purified from the rhizomes of *Dioscorea japonica* Thunb. [Dioscoreaceae](Kim et al., 2011). *Paris polyphylla* Sm. [Melanthiaceae] is one of the SS primary plant sources (Ye et al., 2025). Nie and co-workers isolated a new 12-hydroxyspirostan from *Paris yunnanensis* Franch. (syn. *Paris polyphylla* var. *yunnanensis*). [Melanthiaceae] (Nie et al., 2024). New glucoside SS, including parispolyosides (A-E, Figure 2) with spirostan and cholestane structures, isolated from *Paris chinensis* Franch (syn. *Paris polyphylla* var. *chinensis*). [Melanthiaceae] (Guan et al., 2024). *Paris Steroidal Saponin*s (PSSs), Polyphyllin Steroidal Saponins (PPSSs), parisyunnanosides, parisvaniosides, pennogenins, chonglouosides, and diocin are other SS isolated from different varieties of *P*. *polyphylla* (Thapa et al., 2022; Yan et al., 2022; Jin et al., 2024; Manoochehri et al., 2024; Ye et al., 2025). Notably, several members of the Paris-derived steroidal saponins (e.g., polyphyllins and related derivatives) have been repeatedly reported to suppress phosphorylation of PI3K, Akt, or downstream mTOR targets, highlighting this genus as a particularly relevant source of PI3K/Akt-modulating compounds (He et al., 2019c; Sobolewska et al., 2020; Ahmad et al., 2021). The high diversity and inimitable chemical structures of SS have led to these compounds revealing various biological and pharmacological activities, which are discussed in the following sections. In the context of this review, the structural and botanical diversity described above is presented to frame subsequent sections that specifically dissect how representative SSs from these sources interfere with the PI3K/Akt signaling cascade in cancer models.

## 4 Broad pharmacological profile of SSs as a foundation for PI3K/Akt-targeted anticancer activity

**FIGURE 3:**
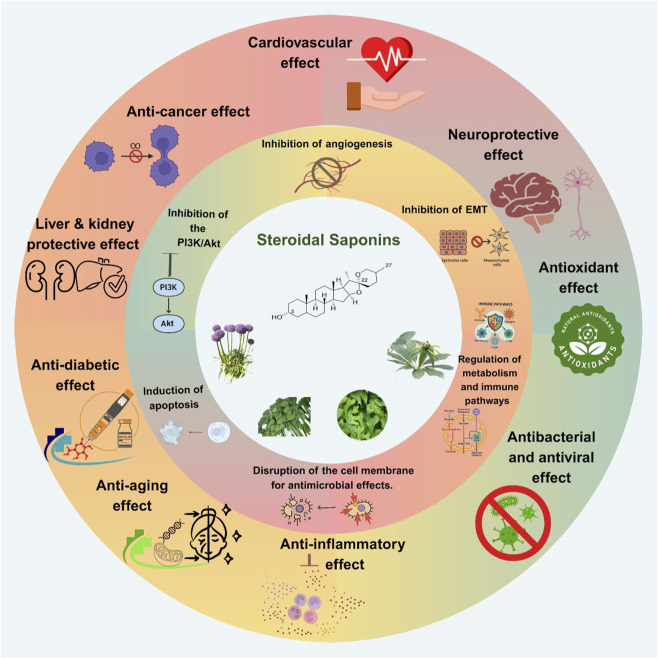
Multifaceted pharmacological effects of steroidal saponins: An overview of their therapeutic activities across systems, including anticancer, cardiovascular, anti-inflammatory, neuroprotective, antioxidant, antidiabetic, hepatoprotective, and antimicrobial properties. Akt: v-Akt murine thymoma viral oncogene homolog, EMT: Epithelial-Mesenchymal Transition, PI3K: Phosphoinositide 3-Kinase.

SSs possess a wide range of biological and pharmacological activities, including anti-inflammatory, antimicrobial, antioxidant, immunomodulatory, anticancer, cardioprotective, hepatoprotective, nephroprotective, neuroprotective, and anti-aging effects. A consolidated overview of these broad activities is provided in Table 1 and Figure 3; however, in line with the scope of this review, the discussion below prioritizes activities with direct relevance to cancer biology and mechanistic intersection with the PI3K/Akt axis (e.g., survival/apoptosis, proliferation, EMT/metastasis, and immune/inflammatory signaling). *Agave marmorata*’s smilagenin-3-*O*-[β-D-glucopyranosyl(1→2)-β-D-galactopyranoside]. The cytotoxicity analysis revealed that NO production was inhibited (EC_50_ = 5.6 mg/mL; E_max_, 101%) and NF-κB reduction was observed (EC_50_ = 0.086 mg/mL; E_max_, 90%) in LPS-stimulated macrophages. Notably, molecular docking suggested a potential PI3K interaction, supporting the concept that selected SSs may interface with upstream components of the PI3K/Akt pathway (Cortés et al., 2022). Diosgenin conjugated with cytarabine (DG-Ara-C) in 116 nm liposomes also showed cytotoxicity (IC_50_ = 35.20 μM HepG2, 50.36 μM MCF-7, and 170.54 μM HL-60) via MTT assays, and better performance than cytarabine as a cancer chemotherapeutic drug (>500 μM) (Liao et al., 2021). Because drug delivery and exposure are key determinants of whether PI3K/Akt modulation translates into *in vivo* efficacy, these formulation-oriented examples are briefly noted here and further discussed in the translational challenges section.

**TABLE 1:** Summary of the biological and pharmacological activities of steroidal saponins.

Activity and therapeutic applications	Key findings	References
Antibacterial	- Diosgenyl 2-amino-2-deoxy-β-D-glucopyranoside activated against *Staphylococcus aureus, Enterococcus faecalis* in BALB/c mice	Weng et al. (2011), Juang and Liang (2020), Shojaee-Aliabadi et al. (2023)
	- Synthetic spirostane saponins are active against Gram-positive bacteria	
Antidepressant	- *Lilium* general effect	Shojaee-Aliabadi et al. (2023)
Antidiabetic	- Diosgenin reduced insulin resistance	Mimaki and Sashida (1996), Barbosa (2014), Sobolewska et al. (2016), Porte et al. (2022)
	- General glycolysis enhancement	
Antifungal/Antiyeast	- Spirostane saponins inhibited *Candida* spp., *Pyricularia oryzae*	Sauvaire et al. (1996), Sautour et al. (2007), Osbourn et al. (2011), Barbosa (2014), Sobolewska et al. (2016), Juang and Liang (2020), Alghirani et al. (2022), Porte et al. (2022)
	- *Allium*: minutesides A–C actives against *Fusarium oxysporum, Alternaria alternata, Botrytis cinerea*; aginoside potent	
	- Avenacins, tomatidine confer fungal resistance	
	- *Paris*: Dioscin damages *Candida albicans*,and *Saprolegnia parasitica*	
	- Fenugreek saponins general claim	
	- Synthetic spirostane saponins active against *Aspergillus niger*	
	- General via cholesterol complexes	
Antiinflammatory	- *Smilax*: sieboldogenin, glycosides reduce PGE_2_, TNFα	Tian et al. (2017), Graziani et al. (2019), Alghirani et al. (2022), Porte et al. (2022), Shojaee-Aliabadi et al. (2023), Tripathi et al. (2023), Gao et al. (2024)
	- *Astragalus*: AS-IV inhibits TLR4/NF-κB	
	Diosgenin inhibits the production of IL-1β, TNF-α, and IL-6	
Antioxidant	- *Astragalus* mitigates lipid peroxidation	Ionkova et al. (2014), Sobolewska et al. (2016), Alghirani et al. (2022), Shojaee-Aliabadi et al. (2023), Gao et al. (2024)
	*Allium* protects H9C2 from H_2_O_2_	
Antithrombotic	- *Allium* furostanol saponins inhibit platelet aggregation	Sobolewska et al. (2016)
Antitumor	- *Ornithogalum* 26, 59% lifespan increased in P388 mice	Price et al. (1987), Mimaki and Sashida (1996), Sautour et al. (2007), Weng et al. (2011), Osbourn et al. (2011), Barbosa (2014), Sobolewska et al. (2016), Tian et al. (2017), Fleck et al. (2019), Graziani et al. (2019), Alghirani et al. (2022), Porte et al. (2022), Shojaee-Aliabadi et al. (2023), Tripathi et al. (2023), Gao et al. (2024), Sun et al. (2025)
	- *Paris*: dioscin, polyphyllin I, TTB2 via PI3K/Akt/mTOR, caspase 9/3	
	- *Dioscorea*: methyl protodioscin, protogracillin, protoneogracillin in xenografts	
	- *Quillaja*: Quil-A prolonged leukemia survival; QS-21 selective apoptosis	
Antiviral	- *Tamus communis*: Dioscin, gracillin weak against VSV, HRV-1B	Slavova et al. (2022)
Cardioprotective	- *Astragalus*: Astragaloside IV protected ischemia/reperfusion via Nrf2, MAPK.	Mimaki and Sashida (1996), Sobolewska et al. (2016), Graziani et al. (2019), Porte et al. (2022), Gao et al. (2024)
	Dioscin prevents cardiac hypertrophy	
	- *Allium* and *Scilla* inhibited cAMP PDE and cardiotonic	
Cytotoxicity	- *Ornithogalum* cholestane glycosides, surpassed etoposide	Price et al. (1987), Mimaki and Sashida (1996), Francis et al. (2002), Sparg et al. (2004), Sautour et al. (2007), Weng et al. (2011), Osbourn et al. (2011), Sobolewska et al. (2016), Tian et al. (2017), Fleck et al. (2019), Graziani et al. (2019), Juang and Liang (2020), Alghirani et al. (2022), Slavova et al. (2022), Porte et al. (2022)
	- OSW-1 via caspase, CREB3–ARF4 pathway	
	- *Paris*: dioscin, polyphyllin I via PI3K/Akt, caspase 8/3 and ets	
	- Diosgenin via mitochondrial pathways; *Tamus* extract outperforms diosgenin	
	- Monodesmosidic saponins enhanced membrane pores, enhancing cytotoxicity	
	- *Quillaja* QS-21 in killing and growth inhibiting (KGI) particles killed tumor cells selectively	
Hepatoprotective	- *Astragalus* mitigated hepatotoxicity	Barbosa (2014), Ionkova et al. (2014), Porte et al. (2022)
	Dioscin inhibits liver necrosis	
	General effect	
Hypocholesterolemic	- Diosgenin reduces LDL via cholesterol complexation	Sauvaire et al. (1996), Weng et al. (2011), Barbosa (2014), Alghirani et al. (2022), Porte et al. (2022)
	Fenugreek binds bile salts	
	- SS’s general effect	
Immunostimulant	- *Astragalus* enhances lymphocyte response	Weng et al. (2011), Barbosa (2014), Ionkova et al. (2014), Alghirani et al. (2022)
	- SSs as vaccine adjuvants	
Neuroprotective (Alzheimer’s treatment)	- *Smilax* enhanced nerve growth factor (NGF) -induced neurite outgrowth	Tian et al. (2017), Graziani et al. (2019)
	- *Astragalus* reduced epilepsy and Alzheimer’s severity	

Akt: Protein kinase B, cAMP PDE: Cyclic adenosine monophosphate phosphodiesterase, HRV-1B: Human rhinovirus 1B, IL-1β: Interleukin-1 beta, IL-6: Interleukin-6, KGI: Killing and growth inhibiting, LDL: Low-density lipoprotein, MAPK: Mitogen-activated protein kinase, mTOR: Mammalian target of rapamycin, NF-κB: Nuclear factor kappa-light-chain-enhancer of activated B cells, Nrf2: Nuclear factor erythroid 2-related factor 2, PI3K: Phosphatidylinositol 3-kinase, PGE_2_: Prostaglandin E2, TLR4: Toll-like receptor 4, TNFα: Tumor necrosis factor-alpha, VSV: Vesicular stomatitis virus.

Zhang et al. also investigated PSSVII isolated from *Trillium tschonoskii* Maxim. [Melanthiaceae] for its breast cancer efficacy. This SS suppressed TNBC (MDA-MB-231 and MDA-MB-468) proliferation via MEK/ERK/STMN1 inhibition, with molecular docking (−8.6 kcal/mol, LYS-97 interaction) and SPR (KD = 1.433 × 10^−5^ M), which confirmed MEK1 binding, and 4T1 xenografts showed 71.26% tumor reduction at 10 mg/kg (Zhang Y. et al., 2024). Dwarf lilyturf tuber (DT-13) from *Liriope muscari* (Decne.) L.H.Bailey [Asparagaceae] inhibited tumor metastasis and improved immunity via EGFR endocytosis and the Src/PI3K/Akt pathways, with 53.58% (40.89% urinary) excretion of metabolites (e.g., deglycosylated ruscogenin) over 8 days (Li et al., 2020).

Rawat et al. investigated the use of *P*. *polyphylla*, dioscin, and diosgenin produced via callus cultures as an antioxidant agent. 2,2′-azinobis(3-ethylbenzothiazoline-6-sulfonic acid) diammonium salt (ABTS), 2,2-diphenyl-1-picrylhydrazyl hydrate (DPPH), and ferric reducing power (FRAP) assays demonstrated the potent antioxidant activity of these components. Their cytoprotective potential is linked to mitigating oxidative stress, suggesting their use in managing chronic inflammatory and degenerative conditions (Rawat et al., 2023).

The anti-inflammatory, hypocholesterolemia, antidiabetic, cardioprotective, and neuroprotective properties of SSs have also been studied for cancer, cardiovascular, and neurological therapies (Sauvaire et al., 1996; Tian et al., 2017; Graziani et al., 2019; Porte et al., 2022).

The anti-inflammatory properties of SSs have been studied. Suresh et al. investigated the anti-inflammatory activity of diosgenin, borassoside D, and govanoside B, which were isolated from *Trillium govanianum* Wall. ex D.Don [Melanthiaceae]. Their results showed significant inhibition of nitric oxide (NO) and pro-inflammatory cytokines (tumor necrosis factor-alpha (TNF-α), interleukin-1 beta (IL-1β), and interleukin-6 (IL-6)) in LPS-stimulated RAW 264.7 macrophages. Diosgenin also exhibited the highest membrane permeability among the compounds studied, suggesting it as a potential anti-inflammatory agent for clinical applications (Suresh et al., 2021). Yang et al. isolated 15 SSs from *Tribulus terrestris* L. [Zygophyllaceae]. These components, with furostanol and isospirostanol backbones, exhibited notable anti-inflammatory activity. Some isolated SSs effectively suppressed TNF-α, while others reduced IL-6 expression in RAW 264.7 cells. Their results also suggested that the yielded SSs yielded novel compounds with chemotaxonomic activity (Yang et al., 2022). Furthermore, anti-diabetic activities of SSs have been investigated. SSs isolated from *Trigonella foenum-graecum* L. [Fabaceae] exhibited potent antidiabetic effects by inhibiting α-glucosidase, with IC_50_ values as low as 5.49 µM. Diosgenin exhibited greater bioavailability and more potent enzyme inhibition than its glycoside forms (Zhang H. et al., 2020). In conclusion, SSs exhibit diverse pharmacological activities (Table 1; Figure 3), but the remainder of this review specifically focuses on mechanistic evidence for how representative SSs modulate PI3K/Akt signaling to impact cancer hallmarks such as survival, proliferation, metastasis, and therapeutic resistance.

## 5 The role of steroidal saponins in cancer therapy

As mentioned in the previous section, SSs have been used to treat the new generation of cancer. Steroidal saponins, with their steroidal backbone attached to sugar moieties, target vital cellular pathways essential to cancer progression. The significant reported anti-tumor activities of SSs are apoptosis, inhibition of tumor growth, and increased sensitivity to chemotherapeutic agents (Lee et al., 2013; Zhang et al., 2015; Wang K. et al., 2023). Furthermore, SSs have been applied along with conventional treatments like chemotherapy and radiation to improve the sensitivity of tumor cells to chemotherapy. The diverse mechanisms of action of SSs, their target pathways, and key findings are summarized in Table 2. This table provides a detailed overview of the induction of apoptosis, inhibition of metastasis, modulation of angiogenesis, and other critical pathways targeted by these compounds. As shown, one of the most notable mechanisms is apoptosis induction, which is often disrupted in cancer cells. In addition to apoptosis, the cell cycle, which is dysregulated in cancer cells, can be influenced by SSs (Wang et al., 2015; Chatterjee et al., 2024). SSs can also inhibit tumor angiogenesis by suppressing the expression of vascular endothelial growth factor (VEGF) and other pro-angiogenic factors (Lazzara et al., 2019; Chien et al., 2022).

**TABLE 2:** Mechanistic overview of steroidal saponins in cancer therapy: target pathways and key findings.

Mechanism	Steroidal saponin	Evidence level/Model/Dose	Target pathway/Effect	Key findings	References
Angiogenesis inhibition	*Helleborus niger* extract	*In vitro* (2D and 3D assays in HUVEC and multiple tumor cell lines); 600–1,000 μg/mL; comparator substances used	VEGF pathway suppression	Reduced angiogenesis in HUVECs and inhibited tumor cell proliferation and migration (>80% inhibition at 600–1,000 μg/mL)	Felenda et al. (2019)
Apoptosis induction	Polyphyllin I steroidal saponin (PPSSI)	*In vitro* (liver cancer cell models) + *In vivo* (tumor-bearing mice); nanoparticle formulation	Oxidative stress, G2/M arrest	Enhanced apoptosis in liver cancer cells with targeted delivery using PEG-CPP44/PPI@IRMOF-8 nanoparticles; improved antitumor efficacy and reduced systemic toxicity	Wang et al. (2023a)
	Paris saponin VII (PSVII)	*In vitro* (TNBC cell lines) + *In vivo* (4T1 xenograft mouse model); intraperitoneal injection, 10 mg/kg; no standard comparator reported	Mitochondrial dysfunction; MEK/ERK/STMN1 signaling suppression	Suppressed TNBC proliferation, migration, and invasion; induced apoptosis; reduced tumor growth by 71.26% *in vivo*	Zhang et al. (2024b)
Cancer stem cell inhibition	Diosgenin	*In vitro* (enriched CSCs from MCF7, T47D, MDA-MB-231 cell lines)	Wnt/β-catenin signaling inhibition (via sFRP4 modulation)	Inhibited breast cancer stem-like cells; induced apoptosis; suppressed β-catenin and EMT-related markers	Bhuvanalakshmi et al. (2017)
Ferroptosis induction	Polyphyllin I steroidal saponin (PPSSI)	*In vitro* (DU145, PC3 CRPC cell lines) + *In vivo* (nude mouse xenograft); dose not specified in abstract; mechanistic comparator: ERK inhibitor	ERK/DNMT1/ACSL4 axis, GPX4 downregulation	Induced ferroptosis in CRPC cells; suppressed tumor growth in nude mouse xenograft model	Zou et al. (2024)
	Timosaponin AIII steroidal saponin (TAIIISS)	*In vitro* (CRC cell lines) + *In vivo* (xenograft tumor model)	Lipophagy-mediated lipid peroxidation (Rab7-dependent); GPX4 and FSP1 downregulation	Induced ferroptosis in colorectal cancer cells and reduced tumor volume in xenograft model	Shen et al. (2024)
Immune modulation	Taccaoside A	*In vitro* (cancer cell lines + T-cell assays) + *Ex vivo* (patient-derived T cells) + *In vivo* (NOG immune-deficient mice)	Activation of mTORC1-Blimp-1 axis in T cells	Enhanced T-cell-mediated tumor killing; extended survival and reduced tumor burden in immune-deficient mouse model	Dai et al. (2022)
Metastasis inhibition	Timosaponin AIII steroidal saponin (TAIIISS)	*In vitro* (human cervical cancer cells + cervical cancer stem cells) + *In vivo* (immunodeficient mouse metastasis model); mechanistic comparator: p38 siRNA	Downregulation of uPA, inhibition of p38 MAPK.	Inhibited migration and invasion in cervical cancer cells and reduced lung metastases in immunodeficient mouse model	Chien et al. (2022)
	Diosgenin	*In vitro* (human prostate cancer PC-3 cell line; wound-healing migration assay, Boyden chamber invasion assay; endothelial tube formation assay); non-toxic concentrations of diosgenin	Suppression of VEGF, MMP-2/MMP-9	Reduced prostate cancer metastasis	Chen et al. (2011)
Multidrug resistance reversal	Asclepiasterol	*In vitro* (P-gp–overexpressing human cancer cell lines: MCF-7/ADR, HepG-2/ADM; 2.5–5.0 µM; combination with paclitaxel or doxorubicin)	P-gp downregulation (protein level); ↓ ERK1/2 phosphorylation (MAPK/ERK pathway)	Increased intracellular drug retention (doxorubicin, Rh123) and enhanced cytotoxicity in MDR cancer cells	Yuan et al. (2016)
Anticancer (cytotoxic activity)	Diosgenyl saponin analogues (synthetic derivatives of dioscin)	*In vitro* (human breast cancer MCF-7 and cervical cancer HeLa cell lines)	Cytotoxicity assessment; structure–activity relationship (monosaccharide vs. disaccharide saponins; acyl substitutions on amino group)	Reduced viability of cancer cells; monosaccharide analogues more cytotoxic than disaccharide analogues	Kaskiw et al. (2009)
Anticancer (pyroptosis induction in PDAC)	Chemical Carcinogenesis Research Information System compounds, Polyphyllin I steroidal saponin, and Paris saponin V	*In vitro* and *in vivo* (human PDAC cell lines PANC-1, AsPC-1, BxPC-3; PANC-1 xenograft in BALB/c nude mice)	Caspase-3 activation; GSDME cleavage (not GSDMD); LDH release; PI staining; flow cytometry	Induction of GSDME-mediated pyroptosis, inhibition of PDAC cell proliferation; CCRIS significantly suppressed tumor growth *in vivo*	Liu et al. (2023a)
Anticancer (growth inhibition and anti-adhesive effect in liposarcoma)	Spirostanol-based F-1 (synthetic steroidal saponin analog)	*In vitro* and *in vivo* (highly malignant human liposarcoma SW872-S cells; xenograft model)	↓ Akt phosphorylation; downregulation of oxysterol-binding protein (OSBP); OSBP knockdown phenocopies SBF-1 effects	Inhibition of liposarcoma cell growth; reduced cell adhesion to fibronectin and laminin; suppression of tumor growth *in vivo*	Chen et al. (2018)
Anti-metastatic and tumor microenvironment modulation (melanoma)	Dioscin	*In vitro* and *in vivo* (murine melanoma B16 cells; macrophage RAW264.7 cells; melanoma metastasis animal models)	Upregulation and functional activation of Connexin-43 (Cx43) via retinoic acid signaling; modulation of CSC and EMT markers; macrophage M2→M1 polarization	Suppressed migration and invasion; reduced EMT and cancer stemness; enhanced pro-inflammatory cytokine release (IL-6, TNF-α, and IL-1β); increased macrophage phagocytosis; significant inhibition of melanoma metastasis *in vivo*	Kou et al. (2017)

ACSL4: Acyl-CoA Synthetase Long Chain Family Member 4, Akt: Protein Kinase B, Blimp-1: B Lymphocyte-Induced Maturation Protein 1, CRC: Colorectal Cancer, CRPC: Castration-Resistant Prostate Cancer, CSC: Cancer Stem Cell, DNMT1: DNA (Cytosine-5)-Methyl transferase 1, EMT: Epithelial–Mesenchymal Transition, ERK: Extracellular Signal-Regulated Kinase, Gasdermin E: A protein that mediates pyroptosis, GSDME: Gasdermin E, GSDMD: Gasdermin D, GPX4: Glutathione Peroxidase 4, HUVECs: Human Umbilical Vein Endothelial Cells, LDH: Lactate Dehydrogenase, MDR: Multidrug Resistance, MMP-2/MMP-9: Matrix Metalloproteinase-2/-9, p38 MAPK: p38 Mitogen-Activated Protein Kinase, mTORC1: Mammalian Target of Rapamycin Complex 1, NSCLC: Non-Small Cell Lung Cancer, OSBP: Oxysterol-Binding Protein, P-gp: P-glycoprotein, PDAC: Pancreatic Ductal Adenocarcinoma, PI: Propidium Iodide, ROS: Reactive Oxygen Species, TNBC: Triple-Negative Breast Cancer, uPA: Urokinase-type Plasminogen Activator, VEGF: Vascular Endothelial Growth Factor, Wnt/β-catenin: Wnt Signaling Pathway/Beta-Catenin.

SSs, as discussed in the previous section, have been applied to study oncogenesis as a new cycle of cancer generation. SSs with a steroid backbone(s) and sugar moieties bind to important cellular targets that are essential to cancer development. The significant reported anti-tumor activities of SSs include apoptosis, tumor growth inhibition, and cellular susceptibility to chemotherapeutic agents. Notably, SSs have also been applied concurrently with conventional treatment therapies, such as chemotherapy or radiation therapy, to improve the sensitivity of tumors to chemotherapeutic agents (Lee et al., 2013; Zhang et al., 2015; Wang K. et al., 2023).

The various mechanisms, effects of SSs, target pathway, and main observations are illustrated in Table 2. This table illustrates the mechanisms (pathways) by which SSs target their effects, and the critical observations pertinent to the induction of apoptosis, inhibition of metastasis, modulation of angiogenesis, and other targets. As illustrated, a key mechanism that induces apoptosis (programmed cell death) in cancer cells is often disrupted in cancer cells. While apoptosis is evident, the cell cycle can also regulate cell growth and division, which are disrupted by SSs (Wang et al., 2015; Chatterjee et al., 2024). The SSs also inhibit tumor angiogenesis by suppressing VEGF expression and other pro-angiogenic factors. SSs also have substantial effects on inhibiting metastasis, which is one of the hallmarks of advanced cancer. Beyond direct antitumor effects, an increasing number of studies show that SSs also cause an increase in cancer cell sensitivity to traditional therapies such as chemotherapy and radiation. Another important aspect of action is the modulation of the immune response (Lazzara et al., 2019; Chien et al., 2022).

Following a comprehensive understanding of the mechanisms, the diverse efficacy of SSs across various cancer types, along with their mechanisms and key outcomes, is summarized in Table 3.

**TABLE 3:** Efficacy of steroidal saponins across specific cancer types.

Cancer type	Steroidal saponin	Mechanism of action	Key findings	Link to PI3K/Akt signaling	References
Breast	Paris saponin VII (PSVII)	MEK/ERK pathway inhibition, caspase activation	Reduced tumor volume in xenografts by 70%	Parallel/crosstalk (MEK/ERK and PI3K/Akt pathways converge on proliferation, survival, and apoptotic regulation in breast cancer cells)	Zhang et al. (2024b)
	Diosgenin	Wnt/β-catenin inhibition, CSC targeting	Reduced breast CSC populations and impaired tumor progression	Indirect (Wnt/β-catenin signaling and cancer stem cell maintenance intersect with PI3K/Akt-dependent survival and self-renewal programs)	He et al. (2014), Bhuvanalakshmi et al. (2017)
	Timosaponin AIII steroidal saponin (TAIIISS)	BMI1 suppression, EMT marker reduction	Inhibited metastasis and enhanced tumor suppression in aggressive breast cancers	Indirect (BMI1-driven stemness and EMT programs are functionally regulated downstream of PI3K/Akt signaling, influencing invasion and metastatic potential)	Gergely et al. (2018)
Castration-resistant prostate	Polyphyllin I steroidal saponin (PPSSI)	NF-κB pathway modulation, ferroptosis induction	Reduced tumor growth with amplified effects when combined with enzalutamide	Parallel/Indirect (NF-κB and ferroptosis-regulating networks exhibit functional crosstalk with PI3K/Akt signaling in CRPC, particularly in survival signaling and resistance to androgen-receptor–targeted therapy)	Xiang et al. (2018), Zou et al. (2024)
Cervical	Timosaponin AIII steroidal saponin (TAIIISS)	p38 MAPK pathway inhibition, metastasis suppression	Reduced migration, metastasis, and lung metastases *in vivo*	Parallel/Indirect (p38 MAPK–dependent control of migration and metastasis operates in parallel with PI3K/Akt signaling, with documented pathway crosstalk regulating cytoskeletal dynamics and metastatic dissemination)	Chien et al. (2022)
	Dioscin	Mitochondrial pathway activation	Induced apoptosis in HeLa cells with an IC_50_ comparable to that of conventional drugs	Indirect (mitochondrial apoptotic signaling is functionally regulated by PI3K/Akt-dependent survival pathways; suppression of Akt activity sensitizes cells to mitochondrial-mediated apoptosis)	Kaskiw et al. (2009)
Colorectal	Deltonin, ZS	Caspase activation, angiogenesis inhibition	Induced apoptosis and reduced angiogenesis in preclinical models	Indirect (PI3K/Akt signaling is a central upstream regulator of caspase-mediated apoptosis and angiogenic processes; attenuation of Akt-dependent survival and VEGF signaling sensitizes colorectal cancer cells to apoptosis and limits tumor angiogenesis)	Tong et al. (2011), Tong et al. (2012)
	Timosaponin AIII steroidal saponin (TAIIISS)	Lipophagy-induced ferroptosis	Reduced tumor volume in xenograft models	Parallel/Indirect (PI3K/Akt/mTOR signaling is a key regulator of lipid metabolism, autophagy, and ferroptosis sensitivity; disruption of Akt-dependent metabolic and redox homeostasis can facilitate lipophagy-driven ferroptotic cell death)	Shen et al. (2024)
	PP9	G2/M arrest, apoptosis	Outperformed 5-fluorouracil in murine colon cancer models	Indirect (PI3K/Akt signaling governs cell-cycle progression and apoptosis resistance; attenuation of Akt-dependent checkpoints and survival signaling can facilitate G2/M arrest and sensitize colorectal tumors to apoptotic cell death)	Yao et al. (2020)
Gastric adenocarcinoma	Shatavarin-IV	EMT inhibition, apoptosis induction	Targeted hyperglycemia-induced gastric cancer	Indirect (hyperglycemia-driven EMT and apoptosis resistance in gastric cancer are tightly regulated by PI3K/Akt/mTOR signaling; suppression of EMT programs and induction of apoptosis functionally converge on attenuation of Akt-dependent metabolic and survival pathways)	Chatterjee et al. (2024)
Liposarcoma	Spirostanol-based F-1 (synthetic steroidal saponin analog)	Akt pathway inhibition, apoptosis induction	Targeted chemoresistant liposarcoma cells and reduced metastasis	Direct (explicit inhibition of Akt phosphorylation leading to suppression of PI3K/Akt-dependent survival signaling, restoration of apoptotic responses, and reduced metastatic potential in chemoresistant liposarcoma cells)	Chen et al. (2018)
Liver	Polyphyllin I steroidal saponin (PPSSI)	Targeted delivery via IRMOF-8, oxidative stress induction	Enhanced therapeutic efficacy with reduced toxicity	Indirect (oxidative stress and survival signaling are tightly regulated by PI3K/Akt; modulation of redox homeostasis can attenuate Akt-dependent cytoprotective responses and sensitize hepatocellular carcinoma cells to apoptosis, while targeted delivery improves effective inhibition of PI3K/Akt-regulated survival pathways *in vivo*)	Wang et al. (2023a)
Lung	Gracillin	Mitochondrial metabolism disruption	Suppressed tumor growth in mutant Kras-driven lung cancers	Indirect (PI3K/Akt/mTOR signaling is a central regulator of metabolic reprogramming and mitochondrial function in Kras-driven tumors; disruption of mitochondrial metabolism functionally converges on attenuation of Akt-dependent bioenergetic and survival signaling, thereby sensitizing lung cancer cells to growth suppression)	Min et al. (2019)
	PEGylated diosgenin	Improved bioavailability and cytotoxicity	Enhanced efficacy in lung cancer models	Indirect (improved bioavailability and systemic exposure enhance functional modulation of PI3K/Akt-regulated survival and apoptotic pathways; increased intracellular delivery of diosgenin facilitates attenuation of Akt-dependent cytoprotective signaling in lung cancer models)	Li et al. (2015a)
Melanoma	Dioscin	Connexin 43-mediated signaling	Reduced metastasis and malignancy	Indirect/Parallel (Connexin-43–mediated suppression of EMT, migration, and malignant progression functionally converges with PI3K/Akt-regulated survival and metastatic programs; PI3K/Akt activity is known to modulate gap-junction integrity, EMT plasticity, and tumor–immune interactions in melanoma)	Kou et al. (2017)
Multidrug-Resistant	Asclepiasterol	P-gp downregulation	Improved drug retention and reduced colony formation in MDR cell lines	Indirect/Parallel (Downregulation of P-glycoprotein reverses multidrug resistance and enhances intracellular drug accumulation; PI3K/Akt signaling is a known upstream regulator of ABC transporter expression and survival signaling in MDR phenotypes, indicating functional convergence)	Yuan et al. (2016)
Pancreatic	Chemical Carcinogenesis Research Information System-derived compounds	Pyroptosis induction	Reduced tumor growth through GSDME activation	Indirect/Parallel (GSDME-mediated pyroptosis counteracts PI3K/Akt-driven survival signaling by promoting caspase-3 activation and inflammatory cell death; functional antagonism exists between Akt-dependent cytoprotective pathways and execution of pyroptosis)	Liu et al. (2023a)
Renal and colorectal	*Helleborus niger* extract	Migration and proliferation inhibition	Reduced cancer cell growth by 80% in preclinical models	Indirect/Parallel (Suppression of cancer cell migration and proliferation reflects functional attenuation of PI3K/Akt-regulated cell-cycle progression, survival, and motility programs; PI3K/Akt signaling is a central upstream driver of these phenotypes in renal and colorectal cancers)	Felenda et al. (2019)

ACSL4: Acyl-CoA Synthetase Long Chain Family Member 4, Blimp-1: B Lymphocyte-Induced Maturation Protein 1, CRPC: Castration-Resistant Prostate Cancer, CSC: Cancer Stem Cell, DNMT1: DNA (Cytosine-5)-Methyltransferase 1, EMT: Epithelial–Mesenchymal Transition, ERK: Extracellular Signal-Regulated Kinase, Gasdermin E: A protein that mediates pyroptosis, GPX4: Glutathione Peroxidase 4, HUVECs: Human Umbilical Vein Endothelial Cells, MDR: Multidrug ResistanceMMP-2/MMP-9: Matrix Metalloproteinase-2/-9, p38 MAPK: p38 Mitogen-Activated Protein Kinase, mTORC1: Mammalian Target of Rapamycin Complex 1, NSCLC: Non-Small Cell Lung Cancer, P-gp: P-glycoprotein, ROS: Reactive Oxygen Species, TNBC: Triple-Negative Breast Cancer, uPA: Urokinase-type Plasminogen Activator, VEGF: Vascular Endothelial Growth Factor, Wnt/β-catenin: Wnt Signaling Pathway/Beta-Catenin.

SSs have been extensively investigated in breast cancer studies. Diosgenin, with significant anticancer effects against breast cancer cell lines (Lazzara et al., 2019; Min et al., 2019), also enhanced the chemosensitivity of breast cancer cells to conventional chemotherapeutic agents by downregulating the expression of multidrug resistance proteins like P-glycoprotein (P-gp) (He et al., 2014; Bhuvanalakshmi et al., 2017). Tumor growth in TNBC xenografts was suppressed by 71% by Paris Steroidal Saponin VII (PSSVII), which targets the MEK/ERK pathway and suppresses key proliferation markers (Zhang Y. et al., 2024). Timosaponin AIII (TAIIISS) and diosgenin exhibited potent inhibitory effects on non-small cell lung cancer (NSCLC) cells by inducing apoptosis and suppressing cell proliferation. TAIIISS has been shown to induce mitochondrial dysfunction in lung cancer cells, leading to caspase activation and subsequent apoptosis (Bhuvanalakshmi et al., 2017; Chien et al., 2022). Diosgenin and timosaponin AIII have also been shown to be highly effective against colorectal cancer (CRC). Diosgenin, in particular, induced apoptosis in CRC cells by modulating apoptosis-related proteins such as Bax and Bcl-2, which activate mitochondrial-dependent cell death. These compounds also exhibit chemopreventive activity by inhibiting tumor formation in CRC model systems (Liu et al., 2013; Bhuvanalakshmi et al., 2017). Gracillin and deltonin have also shown strong activity in colorectal cancer models, through mitochondrial disruption and angiogenesis inhibition, respectively (Tong et al., 2011; Min et al., 2019). Diosgenin has also been found to inhibit the growth of hepatocellular carcinoma cells through apoptosis and to inhibit the NF-κB signaling pathway, which is frequently active in liver cancer and promotes cell survival and proliferation (Liu et al., 2013; He et al., 2014). On the other hand, polyphyllin I has been shown to enhance the anticancer effects of chemotherapy in liver cancer by increasing oxidative stress and DNA damage in HCC cells (Lee et al., 2013; Zhang et al., 2015). SSs showed helpful influences for aggressive and treatment-resistant cancers like pancreatic cancer. Diosgenin has been investigated for its ability to inhibit pancreatic cancer cell proliferation and induce apoptosis. Diosgenin induced apoptosis in pancreatic cancer cells by upregulating pro-apoptotic proteins Bax and cleaved caspases and downregulating anti-apoptotic proteins like Bcl-2 (Chen et al., 2011; Chien et al., 2022). Polyphyllin I induced apoptosis in ovarian cancer cells by activating caspases and modulating mitochondrial dysfunction (Zhang et al., 2015; Chien et al., 2022). Timosaponin AIII has been shown to inhibit the invasion and migration of ovarian cancer cells by altering EMT marker expression, thereby preventing ovarian cancer metastasis and spread (Chatterjee et al., 2024; Wu et al., 2024b). SSs were also effective against cervical and esophageal cancers, as well as ovarian cancer. Their ability to modulate tumor microenvironments, inhibit cell cycle progression, and enhance immune response is under investigation (Lazzara et al., 2019; Min et al., 2019). SSs are often used in conjunction with chemotherapeutic agents to enhance outcomes. Diosgenin has been shown to modulate proteins in the tumor microenvironment, thereby improving the effectiveness of chemotherapy in reducing cancer cell migration and invasion (Chen et al., 2011; Bhuvanalakshmi et al., 2017).

In conclusion, the effect of SSs on particular types of cancer indicates their overall anticancer activity. However, their application will depend on solving the problems of availability, toxicity, and delivery systems (Zhang et al., 2015; Bhuvanalakshmi et al., 2017; Lazzara et al., 2019).

Importantly, the interpretation of the mechanistic and efficacy data summarized in Tables 2, 3 should consider several common sources of experimental bias in the current literature on steroidal saponins. Most evidence is derived from *in vitro* studies using established cancer cell lines, which do not fully capture tumor heterogeneity, immune interactions, or clinical complexity. *In vivo* data are predominantly based on xenograft models in immunodeficient mice, limiting conclusions regarding immune-dependent mechanisms and translational relevance.

Additionally, many studies employ doses or concentrations that may exceed clinically achievable exposure due to the known limitations in bioavailability and pharmacokinetics of steroidal saponins. This issue is addressed in Tables 2, 3 by explicitly annotating evidence level, model system, and dose/exposure. Moreover, the limited use of standard-of-care comparator drugs, such as approved PI3K/mTOR inhibitors, restricts direct benchmarking of efficacy and mechanistic specificity. Finally, variability in experimental design and lack of cross-model validation may affect reproducibility, underscoring the need for standardized models and clinically relevant dosing strategies to strengthen translational interpretation.

## 6 Anti-cancer activities of steroidal saponins through attenuating PI3K/Akt signaling pathway

As previously indicated, SSs exhibit anticancer effects against various malignancies due to their distinctive and diverse chemical structures. These effects are mediated through several mechanisms, including the inhibition of the PI3K/Akt signaling pathway. In this section, evidence was systematically extracted and organized according to the structural class of steroidal saponins, cancer model (*in vitro* and/or *in vivo*), dose or exposure conditions, and primary PI3K/Akt-related molecular readouts, in order to provide a coherent and mechanism-oriented overview rather than a purely descriptive summary.

**FIGURE 4:**
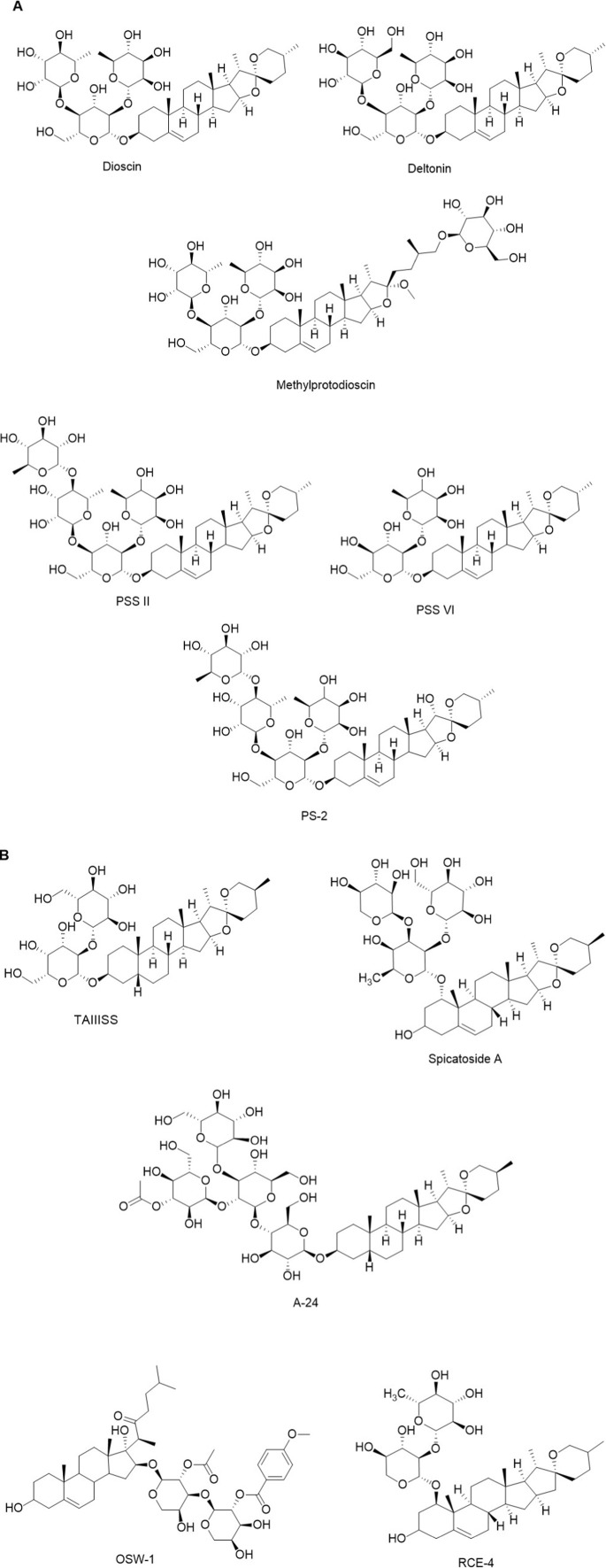
**(A,B)** Chemical structures of some anticancer of steroidal saponins.

Diosgenin and its glycosylated derivatives (dioscin and delatonin), as 3-hydroxyspirostan saponins, are widely distributed in species of the *Dioscorea* and *Allium* genus (Baker et al., 1966; Sobolewska et al., 2016). These compounds have been shown to markedly combat various malignancies, including prostate, lung, colon, ovarian, and bone cancers, by blocking the PI3K/Akt pathway (Chen et al., 2011; Tong et al., 2011; Hsieh et al., 2013; Guo and Ding, 2018; Ruan et al., 2023). Chen et al. stated that inhibition of the PI3K/Akt pathway is one of the main mechanisms of the anti-prostate cancer effect of diosgenin (20 μM) (Chen et al., 2011). Activating autophagy as a vital anti-cancer mechanism by reducing PI3K/Akt activities is one strategy for inhibiting the growth and progression of lung tumor cell lines (H1299 and A549) with dioscin at 2.5 and 5 μM (Hsieh et al., 2013). Additionally, diosgenin exhibited anti-metastatic activity against a bone cancer cell line (MG-6) by inhibiting PI3K/Akt signaling (Ruan et al., 2023). Methyl protodioscin (Figures 4A,B), a diosgenin analog with a furostane base chemical structure, exhibited anti-cancer activities by preventing the proliferation and metastasis of tumor cell lines. Chung et al. reported that methyl protodioscin inhibited cell migration at μM concentration range by blocking the PI3K/Akt pathway (He et al., 2006; Chung et al., 2016). Also, protodioscin prevented bladder tumor progression in mice at 20 mg/kg through the same pathways (Chen et al., 2022).

As mentioned earlier, different varieties of *P*. *polyphylla* are rich natural sources of SSs, including PSSs, PPSSs, and pennogenins (PG), which were mainly purified and isolated from their rhizome. These spirostan SSs exhibit a wide range of anticancer activities, and PI3K/Akt regulation is a key mechanism by which they inhibit tumor progression. *In vivo* and *in vitro* studies indicated that PSSs and PPSSs are potent agents for controlling and treating various types of lung carcinoma by inhibiting the PI3K/Akt pathway (He et al., 2015; He et al., 2019 H.; Liu J. et al., 2016; Liu et al., 2016 Z.; 2017; Zhang L. et al., 2016; Zhu et al., 2016; Yang et al., 2018; Teng et al., 2019). Zhang and co-workers demonstrated that downregulating PI3K/Akt gene expression induced autophagy and cell death in A549 cells treated with PSS II (1 μM) (Zhang L. et al., 2016). Also, the addition of PSS I to two drugs used in lung cancer chemotherapy, camptothecin and its 10-hydroxylated analog, revealed new hope for lung carcinoma treatment. Combining PSS I with these drugs increased inhibition of the PI3K/Akt pathway, resulting in a higher rate of lung tumor cell death (Liu et al., 2017). Additionally, PSSI, II, VI, and VII enhanced the sensitivity of lung cancer cells to chemotherapeutic agents, including gefitinib, a drug used to treat lung carcinoma, through the same signaling pathway (Zhu et al., 2016). Total PSSs (5 mg/kg, orally), by blocking the PI3K/Akt pathway, prevented lung carcinoma induced by urethane (800 mg/kg) in mice (Liu J. et al., 2016). Inhibition of angiogenesis is a critical mechanism by which saponins act as anti-cancer agents (Majnooni et al., 2023). Wang et al. demonstrated that suppressing PI3K/Akt gene expression by PSS I (4 μM) prevented angiogenesis (Wang et al., 2020).

PPSSs I, IV, and VII also induced apoptosis in lung cancer cell lines by downregulating genes in the same pathway, thereby inhibiting tumor progression (Yang et al., 2018; He H. et al., 2019; Teng et al., 2019). PPSS VII demonstrated notable cytotoxic activity against lung tumor cell lines by blocking gene expression in the PI3K/Akt signaling pathway in a time-dependent manner at a concentration of 0.41 μM (He H. et al., 2019). Additionally, suppressing PI3K/Akt gene expression to treat colorectal cancer is another target of PSS VII, PSS II, and PPSS II (Li et al., 2014; 2022; Chen et al., 2019). Intraperitoneal (i.p.) injections of 0.5 and 1 mg/kg PPSS II inhibited PI3K/Akt gene expression and reduced colorectal tumor growth and progression in mice (Li et al., 2022). Other cancers, including breast, brain, liver, and leukemia, were also targeted by PSSs and PPSSs through the suppression of PI3K/Akt gene expression (Zhang C. et al., 2016; Xie et al., 2017; Bi et al., 2021; Liao et al., 2022; Miao et al., 2024; Zhou X. et al., 2024) (Table 1). Miao et al. demonstrated in *in vivo* (0.5 mg/kg/day i.p., mice) and *in vitro* (2.13 μM) studies that PPSS II is a promising candidate for breast cancer treatment by inhibiting PI3k/Akt gene expression (Miao et al., 2024). Furthermore, PPSS I and PPSS VII inhibit liver cancer by blocking the same pathway (Zhang C. et al., 2016; Liao et al., 2022).

PGs are other SSs found in the rhizome of *Paris polyphylla* Sm. [Melanthiaceae] that have revealed anti-cancer activities by obstructing the PI3K/Akt pathway (Chen et al., 2014; Long et al., 2015; Yao et al., 2020; Zhang S. et al., 2020; Thapa et al., 2022; Wu J. et al., 2022). Yao et al., in their study on the cytotoxic activities of a PG (PP-9), reported that PP-9 markedly inhibited colorectal tumor progression in rats at doses of 5 and 10 mg/kg. Additionally, they showed that blocking PI3K/Akt gene expression at 0.75 and 1.5 was the primary anticancer cellular mechanism of PP-9 (Yao et al., 2020). Two PG (PS-1 and PS-2) markedly reduced liver cancer progression in BALB/c nude mice after subcutaneous administration of 3 mg/kg via PI3K/Akt pathway blockade (Chen et al., 2014). N45, as another PG, enhanced sensitivity to temozolomide, a major chemotherapy drug for brain tumors, in temozolomide-resistant glioblastoma cells through the same pathway at 4 μg/mL (Zhang S. et al., 2020).

TAIIISS, as a spirostan SS found in rhizomes of *Anemarrhena asphodeloides* Bunge [Asparagaceae] and stems and roots of *Asparagus* spp. [Asparagaceae] showed potential suppressive effects on signaling pathways related to angiogenesis, apoptosis, metastasis, and inflammation, as well as the PI3K/Akt pathway, thus presenting as a promising candidate for cancer therapy (Zhang F. et al., 2020; Liu Z. et al., 2023). Chiang et al. demonstrated that TAIIISS markedly inhibited PI3K/Akt gene expression at concentrations of 4 and 6 μM, thereby preventing the progression of renal cancer cells (Chiang et al., 2019). In addition, TAIIISS combated leukemia and lung cancer by inducing cell death and increasing sensitivity to chemotherapeutic agents in resistant cancer cells through obstructing the PI3K/Akt pathway (Chen et al., 2016; Zhang et al., 2018; Song et al., 2019; Wang et al., 2019). Two separate studies reported that TAIIISS exhibited cytotoxic activity against T-cell (2 and 8 μM) and myeloid (3, 6, and 9 μM) leukemia cells by suppressing PI3K/Akt gene expression (Zhang et al., 2018; Wang et al., 2019). Blocking the PI3K/Akt pathway was the critical mechanism of TAIIISS anti-neoplastic effects after i.p. administration of 2.5 and 5 mg/kg in a mouse model. Inhibition of this pathway increased sensitivity to taxol, a primary treatment for ovarian and lung carcinomas, in taxol-resistant tumor cell lines (Song et al., 2019). Downregulation of the PI3K/Akt signaling pathway by TAIIISS (20 μM) inhibited growth in pancreatic tumor cells (MarElia et al., 2018).

A-24 is another spirostan saponin, found in bulbs of *Allium chinense* G.Don [Amaryllidaceae], with anti-cancer activities. This SS exhibited marked inhibition of gastric cell growth and migration by downregulating PI3K/Akt gene expression (Xu et al., 2020; 2021).

DT-13 is an anti-cancer SS isolated from tubers of *Ophiopogon japonicus* (Thunb.) Ker Gawl. [Asparagaceae] (common name: *mai tung* or dwarf lilyturf) that prevented the metastasis, migration, angiogenesis, and proliferation in gastric, lung, breast, and prostate cancer cell lines by down-regulating the PI3K/Akt pathway (Zhao et al., 2013; Li et al., 2016; Wang et al., 2018; He et al., 2019b). Li et al. demonstrated that incubation of gastric cancer cell lines with DT-13 for 24 h at 10 and 20 μM reduced PI3K and Akt phosphorylation, thereby inhibiting cancer cell progression (Li et al., 2016). Additionally, DT-13 (5 and 10 μM) inhibited the metastasis of prostate tumor cell lines by blocking the PI3K/Akt pathway (Wang et al., 2018). DT-13 reduced the expression of procollagen-lysine 2-oxoglutarate 5-dioxygenase 2, a critical protein in breast cancer migration and metastasis, by down-regulating the PI3K/Akt pathway (He et al., 2019b).

OSW-1, a cholestane SS found in the bulbs of *Ornithogalum saundersiae* Baker [Asparagaceae], exhibited prominent anti-cancer activities against breast, liver, and brain cancers (Jin et al., 2013; Du et al., 2019; Wu M. et al., 2022; Zhan et al., 2022). Zhan et al. reported that OSW-1 prevented glioma tumor progression after administration of 0.01 mg/kg (i.p.) in BALB/c nude mice by reducing the phosphorylation of PI3K and Akt proteins (Zhan et al., 2022).

RCE-4, a spirostan isolated from the whole plant of *Reineckia carnea* (Andrews) Kunth [Asparagaceae], SSPH I, a spirostan purified from the rhizome of *Tacca plantaginea* (Hance) Drenth (syn. *Schizocapsa plantaginea*) [Dioscoreaceae], spicatoside A, and a spirostan isolated from the tubers of *Liriope muscari* (Decne.) L.H.Bailey (syn. *Liriope platyphylla*)[Asparagaceae], were another SSs that revealed anti-cancer activities through down-regulating the PI3K/Akt pathway (Bai et al., 2016; Kim et al., 2016; Xiang et al., 2019; Zhou et al., 2024a). To integrate these findings across diverse experimental settings, the extracted data are summarized in Table 4 according to cancer type, experimental model, dosage/exposure, and natural source, with a specific focus on studies demonstrating PI3K/Akt pathway inhibition as a primary molecular mechanism.

**TABLE 4:** Anti-Cancer effects of steroidal saponins via PI3K/Akt pathway inhibition: Cancer types, experimental models, and natural sources.

SSs	Cancer types/cell line types	Study type/Dosage	Natural sources	References
Steroidal saponin A-24 isolated from Allium chinense	Gastric cell, Kato-III and AGS	*In vitro*: 4 µM	*Allium chinense* G.Don	Xu et al. (2021)
Steroidal saponin A-24 isolated from Allium chinense	Gastric cell, AGS	*In vitro*: 2.and 4 µM	*Allium chinense* G.Don	Xu et al. (2020)
Steroidal saponin A-24 isolated from Allium chinense	Gastric cell, SGC-7901	*In vitro*: 4 and 8 µM	*Allium chinense* G.Don	Xu et al. (2020)
Delatonin	Colorectal, s SW480, SW620, LOVO, mouse colon cancer cell line C26	*In vitro*, *In vivo*: 10, 20, 40 mg/kg	*Dioscorea* spp. and *Allium* spp.	Tong et al. (2011)
Dioscin	Ovarian, SKOV3	*In vitro*: 1.25, 2.5, 5 μM	*Dioscorea* spp. and *Allium* spp.	Guo and Ding (2018)
Diosgenin	Prostate, PC-3	*In vitro*: 20 μM	*Dioscorea* spp. and *Allium* spp.	Chen et al. (2011)
Dwarf lilyturf saponin 13	Breast, MB-231and MB-468	*In vitro*, *In vivo*: 0.1, 1, and 10 μM	*Ophiopogon japonicus* (Thunb.) Ker Gawl	He et al. (2019b)
Dwarf lilyturf saponin 13	Gastric cell, BGC-823 and A549	*In vitro*: 10 and 20 μM	*Ophiopogon japonicus* (Thunb.) Ker Gawl	Li et al. (2016)
Dwarf lilyturf saponin 13	Prostate, PC3 and DU145	*In vitro*: 2.5, 5, and 10 μM	*Ophiopogon japonicus* (Thunb.) Ker Gawl	Wang et al. (2018)
Dwarf lilyturf saponin 13	Breast, HUVEC	*In vitro*: 0.01, 0.1, and 1 µM	*Ophiopogon japonicus* (Thunb.) Ker Gawl	Zhao et al. (2013)
Dwarf lilyturf saponin 13	Breast, chicken embryo chorioallantoic membranes of 5-day eggs	*In vivo*: 0.001, 0.01, and 0.1 nmol/egg	*Ophiopogon japonicus* (Thunb.) Ker Gawl	Zhao et al. (2013)
Methyl protodioscin	Vascular smooth muscle,A7r5	*In vitro*: 6 μM	*Dioscorea collettii* Hook.f	He et al. (2006), Chung et al. (2016)
Methyl protodioscin	Bladder, non-muscle-invasive 5637, muscle-invasive T24	*In vivo*: 20 mg/kg	*Dioscorea collettii* Hook.f	Chen et al. (2022)
Steroidal saponin N54 isolated from Paris vietnamensis	Glioblastoma, U87, U251,U87R	*In vitro*: 4 μM	*Paris vietnamensis* (Takht.) H.Li	Zhang et al. (2020c)
OSW-1 *Ornithogalum saundersiae* saponin-1	Liver, SK-Hep1	*In vitro*: 100 ng/L	*Ornithogalum saundersiae* Baker	Du et al. (2019)
OSW-1 *Ornithogalum saundersiae* saponin-1	Liver, Hep3B	*In vitro*: 200 ng/nL	*Ornithogalum saundersiae* Baker	Jin et al. (2013)
OSW-1 *Ornithogalum saundersiae* saponin-1	OSW-1 Breast, TNBC; 4T1 and MDA-MB-231	*In vitro*: 0.125 ng/mL (4T1), 6.25 ng/mL (MDA-MB-231); *In vivo* (4T1 mouse): 10 μg/kg i.p. every 3 days; combinations: DOX 5 mg/kg i.v. every 6 days; CBP 8 mg/kg i.p. every 6 days; DOC 10 mg/kg i.v. every 6 days	*Ornithogalum saundersiae* Baker	Wu et al. (2022b)
OSW-1 *Ornithogalum saundersiae* saponin-1	Glioma	*In vitro*: 10 nM	*Ornithogalum saundersiae* Baker	Zhan et al. (2022)
OSW-1 *Ornithogalum saundersiae* saponin-1	Glioma	*In vivo*: 0.01 mg/kg	*Ornithogalum saundersiae* Baker	Zhan et al. (2022)
Pennogenin	Colorectal	*In vivo*: 5 and 10 mg/kg	*Paris polyphylla* Sm	Yao et al. (2020)
*Paris polyphylla* saponin VI	Lung, A549	*In vitro*: 4, 5, and 6 μM	*Trillium tschonoskii* Maxim	Teng et al. (2019)
*Paris polyphylla* saponin VI	Lung, H1299	*In vitro*: 5 and 6 μM	*Trillium tschonoskii* Maxim	Teng et al. (2019)
*Paris polyphylla* saponin VI	Colorectal, HT-29	*In vivo*: 0.75 and 1.5 μM	*Paris lancifolia* Hayata (syn. *Paris polyphylla* var. *latifolia*)	Yao et al. (2020)
*Paris polyphylla* saponin VI	Colorectal, HCT116	*In vivo*: 1.5, 3 μM	*Paris lancifolia* Hayata (syn. *Paris polyphylla* var. *latifolia*)	Yao et al. (2020)
*Paris polyphylla* saponin 9	Liver, HepG2	*In vivo*: 3 mg/kg	*Paris polyphylla* Sm	Chen et al. (2014)
Paris steroidal saponin I	Liver, HepG2	*In vivo*: 3 mg/kg	*Paris polyphylla* Sm	Chen et al. (2014)
Paris steroidal saponin II	Colorectal, HT-29	*In vivo*: 1 μM	*Trillium tschonoskii* Maxim	Li et al. (2014)
Paris steroidal saponin VII	Colorectal, SW-620	*In vivo*: 5 μM	*Trillium tschonoskii* Maxim	Li et al. (2014)
Polyphyllin steroidal saponin II	​	​	​	​
Polyphyllin steroidal saponin VII	Colorectal, HCT116	*In vivo*: 0.5 and 1 mg/kg	Rhizoma Paridis	Li et al. (2022)
*Reineckia carnea* extract compound 4	​	​	​	​
*Paris polyphylla* saponin VI (PPVI)	Lung, A549	*In vitro*: 0.41 μM	*Paris polyphylla* Sm	He et al. (2019a)
*Paris polyphylla* saponin VI (PPVI)	Liver, BALB/c	*In vivo*: 3 mg/kg	Rhizoma paridis	Chen et al. (2014)
*Paris polyphylla* saponin 9	Liver, HepG2 xenografts	*In vivo*: 1 or 3 mg/kg	Rhizoma paridis	Chen et al. (2014)
Paris steroidal saponin I	Cervix, HeLa	*In vitro*: 2.5, 5, and 10 μM	*Reineckia carnea* (Andrews) Kunth	Bai et al. (2016)
Paris steroidal saponin II	Cervix, CaSki	*In vitro*: 12 and 16 μM	*Reineckia carnea* (Andrews) Kunth	Xiang et al. (2019)
Spicatoside A	Colorectal, HCT116	*In vitro*: 1 μM	*Liriope muscari* (Decne.) L.H.Bailey (syn. *Liriope platyphylla*)	Kim et al. (2016)
*Schizocapsa plantaginea* saponin I	Lung, NSCLC (A549 and PC9)	*In vitro*: 0, 0.875, 1.75, 3.5 μM	*Tacca plantaginea* (Hance) Drenth (syn. *Schizocapsa plantaginea*)	Zhou et al. (2024a)
Timosaponin AIII steroidal saponin	Leukemia, Jurkat	*In vitro*: 2 and 8 µM	*Anemarrhena asphodeloides* Bunge and *Asparagus* spp.	Wang et al. (2019), Liu et al. (2023b)
Schizocapsa plantaginea saponin I	Human chronic myelogenous leukemia, K562/ADM	*In vitro*: 1 and 2 µM	*Anemarrhena asphodeloides* Bunge and *Asparagus* spp.	Chen et al. (2016)
Timosaponin AIII steroidal saponin	Leukemia, HL-60	*In vitro*: 3, 6, and 9 µM	*Anemarrhena asphodeloides* Bunge and *Asparagus* spp.	Zhang et al. (2018)
Schizocapsa plantaginea saponin I	Lung, A549 and A2780	*In vitro* and *In vivo*: 2, 4, and 8 µM	*Anemarrhena asphodeloides* Bunge and *Asparagus* spp.	Liu et al. (2023b)
Timosaponin AIII steroidal saponin	Pancreas, PANC-1, BxPC-3	*In vitro*: 5, 10, and 20 µM	*Anemarrhena asphodeloides* Bunge	MarElia et al. (2018)
Schizocapsa plantaginea saponin I	Renal, 786-O and A-498	*In vitro* and *In vivo*: 2, 4, and 8 µM	*Anemarrhena asphodeloides* Bunge and *Asparagus* spp.	Chiang et al. (2019), Liu et al. (2023b)
Timosaponin AIII steroidal saponin	Taxol-resistant cells, A549/Taxol and A2780/Taxol	*In vivo*: 2.5, and 5 mg/kg *In vitro*: 4 and 8 µM	*Anemarrhena asphodeloides* Bunge	Song et al. (2019)

### 6.1 Steroidal saponins (SSs) and derivatives

Cell lines/Cancer models: 5637, T24: Bladder cancer cell lines, 786-O, A-498: Renal cancer cell lines, A549, H1299: Lung cancer cell lines, AGS, Kato-III, SGC-7901, BGC-823: Gastric cancer cell lines, HeLa, CaSki: Cervical cancer cell lines, HepG2: Liver cancer cell line, HT-29, HCT116: Colorectal cancer cell lines, HUVEC: Human umbilical vein endothelial cells (used for angiogenesis studies), Jurkat, K562/ADM, HL-60: Leukemia cell lines, MDA-MB-231, MDA-MB-468: Breast cancer cell lines, NSCLC: Non-small cell lung cancer, PANC-1, BxPC-3: Pancreatic cancer cell lines, PC-3, DU145: Prostate cancer cell lines, SK-Hep1, Hep3B: Liver cancer cell lines, SKOV3: Ovarian cancer cell line, SW480, SW620, LOVO, C26: Colorectal cancer cell lines, U87, U251, U87R: Glioblastoma cell lines. Animal models and Experimental terms: BALB/c: Common laboratory mouse strain used for tumor xenograft models, CAM: Chick embryo chorioallantoic membrane (used in angiogenesis studies), i.p.: Intraperitoneal, xenograft: Transplantation of human tumor cells into mice. Other terms: μM: Micromolar (concentration unit), mg/kg: Milligrams per kilogram of body weight (dose unit), ng/nL, ng/L: Nanograms per nanoliter/liter, Taxol: Paclitaxel, a chemotherapy drug.

Collectively, Table 4 provides a consolidated overview of steroidal saponins that converge on the PI3K/Akt signaling axis across multiple cancer types. Although the experimental models and dosage regimens vary, a consistent pattern emerges in which suppression of PI3K/Akt signaling is associated with reduced proliferation, induction of apoptosis or autophagy, inhibition of angiogenesis and metastasis, and, in some cases, enhanced chemosensitivity. This integrated presentation supports the data extraction strategy outlined in the Abstract and highlights PI3K/Akt inhibition as a shared mechanistic hallmark of structurally diverse steroidal saponins. To improve readability, the main steroidal saponins discussed in this section are summarized in a matrix (Table 5), linking representative compounds to key nodes of the PI3K/Akt network and indicating the direction of effect and evidence level.

**TABLE 5:** Matrix mapping of representative steroidal saponins to PI3K/Akt pathway nodes.

Steroidal saponin	PI3K	Akt (p-Akt)	mTOR/mTORC1	PTEN	VEGF/Angiogenesis	EMT/Metastasis	Apoptosis (BAD/FOXO/Caspase)	Ferroptosis	Evidence level
Spirostanol-based F-1	–	↓ (direct inhibition)	–	–	–	↓	↑ apoptosis	–	*In vitro* + *In vivo*
Polyphyllin I	↓ (indirect)	↓ (indirect)	↓ (functional convergence)	–	–	–	↑ apoptosis	↑ ferroptosis (ERK/DNMT1/ACSL4 axis)	*In vitro* + *In vivo*
Timosaponin AIII	–	↓ (reported in multiple models)	↓ (metabolic convergence)	–	–	↓ (p38/uPA axis)	↑ apoptosis	↑ ferroptosis (Rab7-mediated lipophagy)	*In vitro* + *In vivo*
Diosgenin	–	↓ (functional survival attenuation)	–	–	↓	↓	↑ apoptosis	–	*In vitro*
Dioscin	–	↓ (indirect mitochondrial sensitization)	↓ (reported in some models)	–	↓	↓	↑ apoptosis	–	*In vitro* + *In vivo*
Paris saponin VII	–	↓ (parallel survival suppression)	–	–	–	↓	↑ apoptosis	–	*In vitro* + *In vivo*
Taccaoside A	–	–	↑ mTORC1 activation in T cells	–	–	–	Immune-mediated tumor killing	–	*In vitro* + *Ex vivo* + *In vivo*
Helleborus niger extract	–	–	–	–	↓ VEGF pathway	↓ migration	↑ growth inhibition	–	*In vitro*
Asclepiasterol	–	↓ ERK1/2 (parallel survival axis)	–	–	–	–	Sensitizes to apoptosis	–	*In vitro*

ACSL4: Acyl-CoA Synthetase Long Chain Family Member 4, Akt: Protein Kinase B, BAD: Bcl-2–Associated Death Promoter, DNMT1: DNA (Cytosine-5)-Methyltransferase 1, EMT: Epithelial–Mesenchymal Transition, ERK: Extracellular Signal-Regulated Kinase, FOXO: Forkhead Box O Transcription Factors, mTOR: Mammalian Target of Rapamycin, mTORC1: Mammalian Target of Rapamycin Complex 1, p-Akt: Phosphorylated Akt, PI3K: Phosphatidylinositol 3-Kinase, PTEN: Phosphatase and Tensin Homolog, uPA: Urokinase-Type Plasminogen Activator, VEGF: Vascular Endothelial Growth Factor, ↓: Inhibition/Downregulation, ↑: Activation/Induction, –: No direct evidence reported.

**FIGURE 5:**
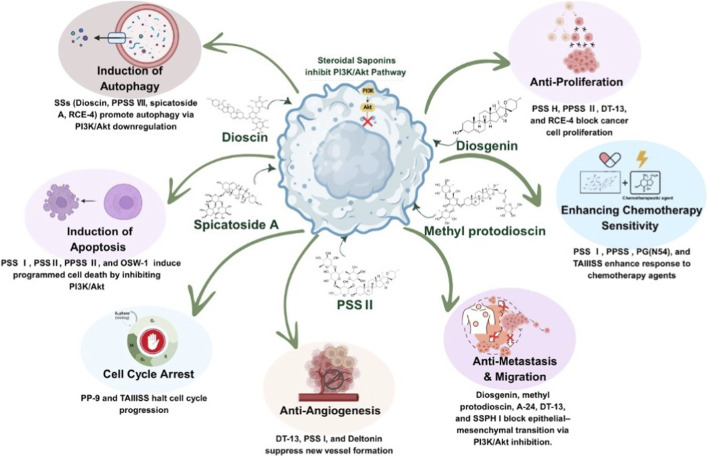
Mechanisms of anticancer activity of steroidal saponins via inhibition of the PI3K/Akt pathway. Abbreviations: A-24: Steroidal saponin isolated from *Allium chinense*, Deltonin: Steroidal saponin derived from *Dioscorea* spp., Dioscin: Steroidal saponin isolated from *Dioscorea* spp. and *Allium* spp., Diosgenin: Steroidal sapogenin derived from *Dioscorea* spp., DT-13: Dwarf lilyturf saponin 13 (from *Liriope muscari*), Methyl protodioscin: Furostane-type steroidal saponin from *Dioscorea collettii*, OSW-1: Cholestane-type steroidal saponin from *Ornithogalum saundersiae*, PG (N54): *Panax notoginseng* fraction N54, PP-9: Polyphyllin 9 (a steroidal saponin derivative), PPSS: Polyphyllin steroidal saponin, PSS: Paris Steroidal Saponin, RCE-4: *Reineckia carnea* extract compound 4, Spicatoside A: Steroidal saponin isolated from *Liriope platyphylla*, SSPH I: *Schizocapsa plantaginea* saponin I, SSs: Steroidal saponins, TAIIISS (TAIII): Timosaponin AIII—steroidal saponin mainly from *Anemarrhena asphodeloides* and some *Asparagus* spp.

To unify the various effects of saponins on anticancer therapy, Figure 5 illustrates how SSs might inhibit the PI3K/Akt pathway to trigger key anticancer effects, including increased autophagy, decreased apoptosis, blocked cell cycle progression, and prevention of angiogenesis, metastasis, and tumor growth. Some SSs even sensitize cancer cells to chemotherapy. These mechanisms, together with the initial example of apoptotic induction, exemplify the potent and simultaneous action of SSs in mitigating cancer progression.

## 7 Preclinical and emerging evidence

Studies have shown that SSs exhibit potent anticancer activity across cancer types and specific pathways. These studies have demonstrated that using these compounds reduces the risk of drug resistance.

Polyphyllin I showed synergistic anticancer activity when combined with enzalutamide for the treatment of castration-resistant prostate cancer (CRPC). This combination downregulated HOTAIR and NF-κB signaling (Xiang et al., 2018). Similarly, using TAIIISS-enhanced siRNA targeting p38 MAPK in cervical cancer cells reduced invasion and migration (Chien et al., 2022).

Radiotherapy outcomes have also been improved by incorporating SSs into the treatment regimen. Diosgenin enhanced tumor cells’ sensitivity to chemoradiation, thereby reducing the required dose (Li C. et al., 2015; Felenda et al., 2019).

Dai et al. investigated the immunomodulatory activity of SSs, when combined with the immune checkpoint inhibitor taccaoside A. The results showed that taccaoside A boosted T-cell cytotoxicity against tumors (Dai et al., 2022).

SSs can also overcome multidrug resistance (MDR), a significant problem in cancer therapy. Asclepiasterol increased the intracellular accumulation of chemotherapeutic agents, such as doxorubicin and paclitaxel, by reversing the function of P-gp, a MDR-associated transporter (Yuan et al., 2016). Moreover, polyphyllin II enhanced the effectiveness of chemotherapy in colorectal cancer by modulating glycolysis and oxidative stress, providing a novel approach to targeting energy metabolism in cancer cells (Wu et al., 2024b).

SSs also demonstrated the potential to interact with novel therapeutic strategies, such as pyroptosis and ferroptosis, at high efficiency (Liu Y. et al., 2023; Shen et al., 2024). Pyroptosis is an inflammatory form of programmed cell death characterized by the activation of inflammatory caspases and the formation of membrane pores, leading to cell swelling and lysis (Liu Y. et al., 2023). Ferroptosis, by contrast, is a non-apoptotic form of programmed cell death driven by iron-dependent lipid peroxidation and oxidative stress. TAIIISS was discovered to promote Rab7-mediated lipophagy, leading to lipid peroxidation and ferroptosis, thereby sensitizing human colorectal cancer cells to treatment (Shen et al., 2024).

These preclinical studies highlight the potential of SSs as monotherapies or in combination with other agents for the treatment of various cancers.

## 8 Challenges, limitations, and future perspectives

SSs in cancer therapy have been promising, but several factors limit their clinical application. These problems, primarily related to bioavailability, systemic toxicity, and target specificity, must be addressed before they can be realized. The most significant challenge of SSs is their poor bioavailability, primarily due to low solubility and a short circulation half-life (Li C. et al., 2015; Zhang et al., 2015; Wang K. et al., 2023). For example, diosgenin cannot be readily absorbed into the bloodstream because it is rapidly metabolized and unstable in the body (Felenda et al., 2019; Teymouri and Karimi, 2024). To address this, novel drug-delivery approaches, including nanoparticle encapsulation and PEGylation, have been developed. These nanoparticle formulations enhance the solubility and stability of saponins, providing sustained release to improve their activity (Liu et al., 2013; Li C. et al., 2015). These approaches include isoreticular metal-organic framework (IRMOF-8)-based nanoparticles, developed to encapsulate PPSSI and address bioavailability issues, thereby reducing side effects (Wang K. et al., 2023). Liposomal and chitosan-folate nanoparticles for diosgenin have enhanced the drug’s stability and targeted delivery (Li C. et al., 2015; Teymouri and Karimi, 2024). Similarly, PEGylated diosgenin nanoparticles co-delivering hydroxycamptothecin (HCPT) have demonstrated improved therapeutic outcomes in lung cancer models by enhancing HCPT bioavailability and reducing its side effects (Li C. et al., 2015). These developments illustrate the application of nanotechnology to improve bioavailability. To better contextualize these translational barriers and avoid a purely descriptive listing, Table 6 maps each major limitation of steroidal saponins to actionable strategies, their expected benefits, and representative supporting evidence. Despite these encouraging preclinical advances, the clinical translation of steroidal saponins remains limited, partly due to pharmacokinetic mismatches between experimental animal models and humans. In rodent studies, SSs are frequently administered at relatively high doses or via optimized delivery systems that can transiently compensate for poor solubility and rapid clearance. In contrast, in humans, extensive first-pass metabolism, rapid biotransformation, and short systemic half-life markedly reduce effective drug exposure at tumor sites. Moreover, species-dependent differences in metabolic enzyme activity, plasma protein binding, and membrane composition may permit apparent efficacy in murine models while simultaneously increasing toxicity risk or diminishing therapeutic benefit in clinical settings. Collectively, these pharmacokinetic and interspecies discrepancies represent a major translational barrier and help explain why many promising preclinical outcomes of SSs have not yet translated into successful human trials (Li X. et al., 2015; Zhang et al., 2015; Felenda et al., 2019; Wang K. et al., 2023; Teymouri and Karimi, 2024).

**TABLE 6:** Strategic mapping of translational limitations of steroidal saponins to actionable solutions.

Translational limitation	Main issue	Proposed strategy	Expected improvement
Poor bioavailability	Low solubility, rapid clearance	PEGylation	Increased stability and circulation time
	Physicochemical instability	Nanoparticle delivery	Improved solubility and sustained release
	Non-specific distribution	Liposomal/polymeric formulations	Enhanced tumor delivery and stability
Systemic toxicity	Non-selective membrane effects	Controlled-release formulations	Reduced off-target toxicity
	High effective doses	Combination therapy	Dose reduction via pathway sensitization
Limited specificity	Cholesterol-dependent interactions	Structural modification/prodrugs	Improved selectivity
	Poor tumor targeting	Targeting ligands (e.g., folate)	Enhanced tumor uptake

The non-specific distribution or high doses of some SSs may exhibit adverse effects on healthy cells and normal tissues, potentially leading to hemolysis (Chen et al., 2011; Tong et al., 2012). Mechanistically, the systemic toxicity and hemolytic activity of steroidal saponins are closely associated with their physicochemical interactions with membrane cholesterol. Structural features of SSs, including the aglycone backbone and the number and position of glycosidic moieties, critically determine their affinity for cholesterol-rich lipid domains, leading to membrane destabilization, pore formation, and loss of membrane integrity. This cholesterol-dependent membrane disruption is well recognized as the primary cause of saponin-induced hemolysis and contributes to their limited target specificity, as such interactions are not restricted to cancer cells. While membrane perturbation may enhance cytotoxic efficacy against tumor cells and synergize with PI3K/Akt pathway inhibition, uncontrolled systemic exposure narrows the therapeutic window and increases the risk of off-target toxicity. Accordingly, rational structural modification and formulation-based strategies are required to mitigate cholesterol-driven membrane toxicity while preserving the anticancer potential of SSs (Sparg et al., 2004; Chen et al., 2011; Osbourn et al., 2011; Sobolewska et al., 2016; Porte et al., 2022).

For instance, the extract of *Helleborus niger* L. [Ranunculaceae] (HNE) is relatively non-toxic at an optimal concentration, provided proper drug administration protocols are followed (Felenda et al., 2019). This controlled drug-delivery system can include liposomal formulations and cell-penetrating peptides (CPP44), which are being investigated to improve tumor targeting and reduce side effects in normal tissues (Li C. et al., 2015; Wang K. et al., 2023; Chatterjee et al., 2024). Polyphyllin II influences glycolytic metabolism by targeting specific pathways and has been found to reduce side effects while improving treatment outcomes (Shen et al., 2024; Wu et al., 2024b). Another challenge is standardizing natural products containing SSs. Variations in extraction techniques and plant sources can affect efficacy and safety. For instance, the extract of *Olax subscorpioidea* Oliv. [Olacaceae] require standardization to ensure consistent therapeutic effects (Popoola et al., 2016). The stability and delivery of SSs in the systemic circulation were also cited as challenges. Many SSs, such as OP-D and dioscin, are unstable in biological systems, which limits their therapeutic efficacy (Kou et al., 2017; Lee et al., 2018). New formulations can enhance the stability and delivery of SSs to the target site (Zheng et al., 2016; Jiang et al., 2022).

**FIGURE 6:**
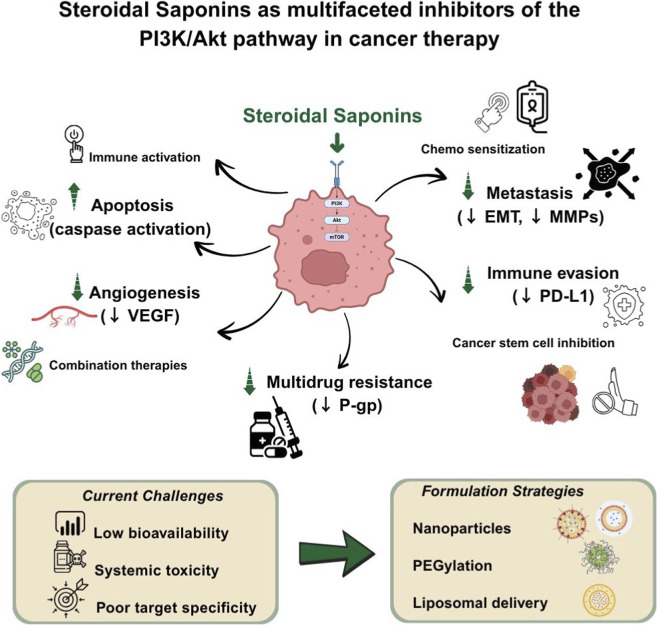
Challenges, limitations, and future perspectives of SSs as inhibitors of the PI3K/Akt in cancer therapy. Akt: v-Akt murine thymoma viral oncogene homolog, EMT: Epithelial-Mesenchymal Transition, MMPs: Matrix Metalloproteinases, mTOR: Mechanistic Target of Rapamycin, PD-L1: Programmed Death-Ligand 1, P-gp: P-glycoprotein, PEGylation: Polyethylene Glycol conjugation, PI3K: Phosphoinositide 3-Kinase, VEGF: Vascular Endothelial Growth Factor.

Regarding the related challenges of SSs, more research should focus on improving the formulations of these compounds to enhance their stability, solubility, and targeting properties. SSs are important therapeutic candidates that significantly influence resistance pathways (Zheng et al., 2016; Zou et al., 2024). Further research on the extraction and formulation of these compounds is likely to lead to their clinical application and may bring about a positive impact on cancer management (Table 6; Figure 6).

## 9 Conclusion

The PI3K/Akt pathway regulates cancer cell growth, survival, angiogenesis, metabolism, and immune evasion. Dysregulation of the pathway, often due to changes at the genetic level, such as PIK3CA mutations or PTEN loss, underlies most cancers, such as breast, prostate, lung, and colorectal cancer. Targeted agents such as alpelisib and everolimus have been shown to be clinically effective in pathway inhibition (Schwartz, 2024; Siegel et al., 2024), but resistance pathways (i.e., pathway reactivation or compensatory signaling via the MAPK pathway) have short-circuited their long-term effectiveness (Mattioli et al., 2023). The validity of developing new therapeutic strategies to bypass this process is therefore required. SSs have been recognized as promising candidates for response molecules in cancer treatment because they can target multiple cancer hallmarks simultaneously. SSs with diverse chemical structures can exhibit various anticancer activities, including induction of apoptosis, inhibition of angiogenesis and metastasis, immune modulation, reversal of multidrug resistance, and interference with cancer cell metabolism (Du et al., 2023; Xu et al., 2023). As noted above, numerous SSs, such as diosgenin, dioscin, polyphyllins, and TAIIISS, contribute to their mechanisms of action by downregulating the PI3K/Akt signaling pathway, thereby affecting tumor growth and progression. Preclinical evidence also indicates that SSs can enhance the effects of chemotherapy and radiotherapy by sensitizing cancer cells, promoting immunogenic cell death, and targeting cancer stem cells. SSs’ modulatory effects on immune checkpoints can bolster T-cell activation and cytotoxicity, further supporting their use in integrated immunotherapy (Chatterjee et al., 2024; Wu et al., 2024b). Moreover, SSs have shown potential for reversing multidrug resistance, a significant barrier to effective cancer therapy, by downregulating efflux proteins, such as P-gp. Although these results are encouraging, the clinical relevance of SSs is still hindered by insufficient bioavailability, high clearance rates, and a potential for toxicity to normal tissues (Sparg et al., 2004; Felenda et al., 2019; Fleck et al., 2019). New formulations, such as nanoparticle encapsulation, PEGylation, or liposome delivery, have shown potential to enhance solubility, stability, and reduce toxicity, with some targeting methods. Further studies on developing delivery systems and standardizing extraction methods to enable reproducible materials would continue to advance SS’s anticancer applications. Future directions should not focus solely on SSs as a single treatment regimen but also on combining SSs with other therapies to explore multiple oncogenic pathways, thereby enhancing their potential synergy with chemotherapeutic agents or immunotherapies. Future studies would benefit from preclinical and clinical studies that define the safety, efficacy, and dosing regimens of SSs. Further studies would benefit from understanding the mechanisms of action of SSs in modulating the PI3K/Akt signalling pathway and their synergistic effects on other signaling networks, to better inform the design of precision oncology therapies.

In conclusion, SSs represent a novel class of natural products with considerable potential in cancer therapy. They offer a variety of targets and approaches to inhibit the PI3K/Akt signaling pathway and its downstream events. In the context of formulation science and clinical research, these compounds have the potential to be used in cancer treatment with reduced or no toxicity.

## Glossary

μM: Micromolar (concentration unit)
5637, T24: Bladder cancer cell lines
786-O, A-498: Renal cancer cell lines
A549, H1299: Lung cancer cell lines
ABTS: 2,2′-azinobis-(3-ethylbenzothiazoline-6-sulfononic acid) diammonium salt
ACSL4: Acyl-CoA synthetase long chain family member 4
AGS, Kato-III, SGC-7901, BGC-823: Gastric cancer cell lines
BAD: Bcl-2-associated death promoter
Alpelisib: PI3K inhibitor
Akt: Protein kinase B
BALB/c: Common laboratory mouse strain used for tumor xenograft models
Blimp-1: B lymphocyte-induced maturation protein 1
CAM: Chick embryo chorioallantoic membrane (used in angiogenesis studies)
cAMP PDE: Cyclic adenosine monophosphate phosphodiesterase
COL1A1: collagen 1A1
CRPC: Castration-resistant prostate cancer
CSC: cancer stem cell
DENV-2: Dengue virus serotype 2
DNMT1: DNA (cytosine-5)-methyl transferase
DPPH: 2,2-diphenyl-1-picryl-hydrazyl-hydrate
1EMT: Epithelial-mesenchymal transition
EOC: epithelial ovarian cancer
ERK: Extracellular signal-regulated kinase
Everolimus: mTOR inhibitor
FGFR1: fibroblast growth factor receptor 1
FOXO: Forkhead box O
FRAP: ferric reducing power
Gasdermin E: A protein that mediates pyroptosis
GPX4: Glutathione peroxidase 4
HeLa, CaSki: Cervical cancer cell lines
HepG2: Liver cancer cell line
HER2: Human epidermal growth factor receptor 2
HPLC: High-performance liquid chromatography
HRMS: High-resolution mass spectrometry
HRV-1B: Human rhinovirus 1B
HT-29, HCT116: Colorectal cancer cell lines
HUVECs: Human umbilical vein endothelial cells
IL-1β: Interleukin-1 beta
IL-6: Interleukin-6
i.p.: Intraperitoneal
JAK/STAT: Janus kinase/Signal transducer and activator of transcription
Jurkat, K562/ADM, HL-60: Leukemia cell lines
KGI: Killing and growth inhibiting
LDL: Low-density lipoprotein
LINC00470: Long intergenic non-coding RNA 00470
LPS, LY3023414: samotolisib
MAPK: Mitogen-activated protein kinase
MC-LR: Microcystin-LR
MDA-MB-231, MDA-MB-468: Breast cancer cell lines
MDR: Multidrug resistance
mg/kg: Milligrams per kilogram of body weight (dose unit)
MMP-2/MMP-9: Matrix metalloproteinase-2/-9
MTHFD2: Methylenetetrahydrofolate dehydrogenase 2
mTOR: Mammalian target of rapamycin
mTORC1: Mechanistic target of rapamycin complex 1
mTORC2: Mechanistic target of rapamycin complex 2
MTT: tetrazolium salt assay
NF-κB: Nuclear factor kappa-light-chain-enhancer of activated B cells
ng/nL, ng/L: Nanograms per nanoliter/liter
NMR: nuclear magnetic resonance
NO: nitric oxide
Nrf2: Nuclear factor erythroid 2-related factor 2
NSCLC: Non-small cell lung cancer
p38MAPK: p38 Mitogen-activated protein kinase
p53: tumor protein p53
PANC-1, BxPC-3: Pancreatic cancer cell lines
PC-3, DU145: Prostate cancer cell lines
PDE: Cyclic adenosine monophosphate phosphodiesterase
PD-L1: Programmed death-ligand 1
PEGylation: Polyethylene glycol conjugation
**PGE**_**2**_: Prostaglandin E2
P-gp: P-glycoprotein
PI3K: Phosphatidylinositol 3-kinase
PIK3CA: p110α subunit of PI3K
PIP2: Phosphatidylinositol 4,5-bisphosphate
PIP3: Phosphatidylinositol 3,4,5-trisphosphate
PTEN: Phosphatase and tensin homolog (tumor suppressor)
PXMP4: Peroxisomal Membrane Protein 4
ROS: Reactive oxygen species
RTKs: Receptor tyrosine kinases
TGCTs: testicular germ cell tumors
TNFα: Tumor necrosis factor-alpha
SK-Hep1, Hep3B: Liver cancer cell lines
SKOV3: Ovarian cancer cell line
SSs: Steroidal saponins
SW480, SW620, LOVO, C26: Colorectal cancer cell lines
TGCT: Testicular germ cell tumor
TIPE2: tumor necrosis factor α-induced protein-8-like-2
TLC: thin-layer chromatography
TLR4: Toll-like receptor 4
TNBC: Triple-negative breast cancer
TNFα: Tumor necrosis factor-alpha
TSAIII/TA-III: Timosaponin AIII
U87, U251, U87R: Glioblastoma cell lines
uPA: Urokinase-type plasminogen activator
VEGF: Vascular endothelial growth factor
VSV: Vesicular stomatitis virus
Wnt/β-catenin: Wnt Signaling pathway/beta-catenin
Xenograft: Transplantation of human tumor cells into mice
ZEB1: Zinc finger E-box-binding homeobox
